# Diagnostic Biomarkers and Targeted Drug Prediction for Acute Kidney Injury: A Computational Approach

**DOI:** 10.2174/0118715303417142250724042300

**Published:** 2025-07-30

**Authors:** Liuyin Zhou, Lian Pan, Jiayang Gao, Yi Jiang, Tingting Li, Ruoqing Li

**Affiliations:** 1 Department of Respiratory Medicine, Chongqing University Central Hospital, Chongqing Emergency Medical Center, Chongqing Key Laboratory of Emergency Medicine, Chongqing, 400014, China;; 2 Department of Plastic Surgery, Chongqing University Central Hospital, Chongqing Emergency Medical Center, Chongqing Key Laboratory of Emergency Medicine, Chongqing, 400014, China;; 3 Department of Biopharmaceutical, College of Food Science and Technology, Shanghai Ocean University, Shanghai, 200090, China;; 4 Department of General Medicine, Chongqing University Central Hospital, Chongqing Emergency Medical Center, Chongqing Key Laboratory of Emergency Medicine, Chongqing, 400014, China

**Keywords:** Acute kidney injury, diagnostic biomarkers, transcription factors, drug prediction, molecular docking, modular genes

## Abstract

**Introduction:**

Acute Kidney Injury (AKI) is a clinical syndrome with rapid onset and poor prognosis, and existing diagnostic methods suffer from low sensitivity and delay. To achieve early identification and precise intervention, there is an urgent need to discover new precise biomarkers.

**Methods:**

AKI samples were acquired from Gene Expression Omnibus (GEO) database. AKI-related module genes were identified using the “WGCNA” package. The “Limma” package was used to filter Differentially Expressed Genes (DEGs). Protein interaction networks were constructed by intersecting key modular genes with DEGs, and six algorithms (MCC, MNC, Degree, EPC, Closeness, and Radiality) in the cytoHubba plug-in were combined to screen candidate genes. Diagnostic biomarkers were cross-screened using LASSO regression with Support Vector Machine–Recursive Feature Elimination (SVM-RFE) machine learning algorithm, and their predictive performance was verified by Receiver Operating Characteristic (ROC) analysis. Transcription Factors (TFs) regulatory network was constructed applying Cytoscape 3.8.0. Finally, the prediction and molecular docking analysis of potential target drugs were performed using the DSigDB database and AutoDockTools.

**Results:**

A total of 498 key modular genes significantly associated with AKI were screened, and 88 AKI-related DEGs and 18 candidate genes were further identified. Importantly, four biomarkers with high diagnostic value *(DDX17, FUBP1, PABPN1,* and *SF3B1*) were screened and validated using dual machine learning algorithms, including LASSO regression and SVM-RFE. The area under the ROC curve (AUC) values for these biomarkers were greater than 0.8, indicating good predictive performance. Moreover, 19 TFs and 17 miRNA of *SF3B1*, 10 TFs and 58 miRNA of *PABPN1*, 15 TFs and 60 miRNA of *FUBP1*, together with 13 TFs and 109 miRNA of *DDX17,* were screened. Drug prediction and molecular docking analysis revealed that Demecolcine and Testosterone Enanthate stably bind to certain markers.

**Discussion:**

Four potential biomarkers closely related to AKI were identified, which may be involved in the occurrence and progression of AKI by regulating key processes such as transcription. The predicted Demecolcine and Testosterone Enanthate may also be involved in the repair of renal injury by regulating key target genes. Although further experimental validation is still needed, these may still provide new intervention strategies for the treatment of AKI.

**Conclusion:**

To conclude, four AKI biomarkers with high diagnostic value were screened by integrating multiple computational methods, revealing a new perspective on the molecular mechanism of AKI. The results provided a new theoretical basis for achieving early precision diagnosis and individualized treatment of AKI.

## 1 INTRODUCTION

Acute Kidney Injury (AKI) is a severe clinical syndrome characterized by complex pathophysiological mechanisms [1-3]. The complications of AKI include renal acidosis, organ edema, extrarenal organ damage, and other Chronic Kidney Disease (CKD), posing a huge threat to human health [4]. AKI is strongly associated with increased in-hospital mortality, particularly among critically ill patients [5], with reported death rates as high as 50-65.2% [6, 7]. At present, AKI diagnosis relies on a sharp decline in glomerular filtration rate, as indicated by elevated Serum Creatinine (SCr) or reduced urine output over a defined period [8]. However, SCr levels in patients often rise gradually and remain initially asymptomatic, leading to delayed AKI diagnosis even when applying the latest guideline of Kidney Disease: Improving Global Outcomes (KDIGO) [9]. Early AKI detection is crucial for timely intervention and prevention of adverse outcomes such as renal dysfunction induced by AKI-related complications [10]. Therefore, discovering novel sensitive and accurate biomarkers that precede measurable SCr elevation has become a crucial research challenge to optimize therapeutic strategies and improve prognosis for patients with AKI.

In recent years, computational analysis such as WGCNA and machine learning algorithms have been widely employed to discover potential disease-associated biomarkers [11, 12], contributing to the development of personalized medicine. WGCNA can be used to examine the connection between critical genes and phenotypes by clustering genes with similar expression patterns, thereby allowing deep learning analysis and precise clinical assessment [13, 14]. Machine learning techniques, such as LASSO regression and SVM-RFE analysis, can efficiently analyze massive genomic data to identify critical diagnostic genes involved in various diseases [14, 15]. In recent years, researchers have extensively explored the molecular mechanisms and early prediction tools of AKI through high-throughput data and machine learning techniques. In one study, key genes such as *GBP2*, *PSMB8,* and *PSMB9* were identified and verified to be closely related to the immune-inflammatory pathway, showing good diagnostic potential in animal models [16]. Another study constructed predictive models with interpretability based on multiple retrospective and prospective cohorts, all of which showed better predictive performance than traditional biomarkers in multicenter validation. These studies emphasized the promising applications of bioinformatics and artificial intelligence in AKI diagnosis and risk stratification [17], highlighting the potential of bioinformatics and machine learning algorithms for diagnosing AKI and risk stratification. Compared with the above studies focusing on the construction of specific clinical types or risk prediction models, the present study systematically screens diagnostic markers and combines them with molecular docking to predict targeted drugs by integrating co-expression network analysis and dual machine learning algorithms, realizing for the first time a one-stop analysis from molecular identification to intervention strategies. In our present study, an AKI-related dataset was collected from the GEO database. Key module genes associated with AKI were identified using WGCNA, and functional enrichment analysis was conducted to elucidate the involved pathways. Candidate genes were screened by PPI network analysis. Additionally, the diagnostic biomarkers with a strong predictive performance in AKI were selected by LASSO regression and SVM-RFE analysis. Furthermore, the TFs of the biomarkers and targeted drugs for AKI were predicted. The current discoveries could enhance the understanding of AKI, providing novel insights for the early diagnosis and personalized management of AKI patients.

## 2 MATERIALS AND METHODS

### 2.1 Data Source

The dataset GSE61739 (https://www.ncbi.nlm.nih.gov/geo/query/acc.cgi?acc=GSE61739), which contained one-month formalin-fixed paraffin-embedded biopsies from 48 kidney transplant recipients (24 AKI samples and 24 control samples), was retrieved from the GEO database.

### 2.2 Wgcna

Critical module genes associated with AKI were screened using the 'WGCNA' package [18]. Briefly, samples were clustered to detect and eliminate the outliers. Based on the intercept height (R^2^) > 0.9, the optimal soft threshold was determined to establish a scale-free topology network. Subsequently, the gene co-expression modules with a minimum gene number of 100 and a merging height of 0.2 were obtained. Utilizing Spearman method, the module-trait relationship was analyzed to identify the key module showing the strongest correlation with AKI. After removing unidentifiable genes, the critical module genes were retained for subsequent analysis.

### 2.3 Functional Enrichment Analysis

The critical module genes identified by WGCNA were uploaded into the KOBAS-i database (http://bioinfo. org/kobas), followed by the use of the “clusterProfiler” R package to conduct Gene Ontology (GO) and Kyoto Encyclopedia of Genes and Genomes (KEGG) enrichment analysis [19]. Molecular Function (MF), Biological Process (BP), and Cellular Component (CC) were the three terms studied in GO analysis. Significantly enriched GO terms and KEGG pathways were screened under *p*<0.05.

### 2.4 Analysis on the Differentially Expressed Genes (Degs)

The DEGs between the AKI samples and control samples in GSE61739 dataset were screened by the “Limma” R package (*p*<0.01) [20] and visualized into a volcano plot. Thereafter, the DEGs were intersected with the critical module genes to acquire AKI-related DEGs for further study.

### 2.5 PPI Network Analysis

The AKI-related DEGs were loaded into the STRING (https://cn.string-db.org/) database to develop a PPI network, which was visualized by cytoscape3.8.0 software [21]. Afterwards, the candidate genes related to AKI were screened by intersecting the top 20 significant genes identified by the 6 algorithms of cytoHubba plug-in, including Maximal Clique Centrality (MCC), Maximum Neighborhood Component (MNC), Degree, Edge Percolated Component (EPC), Closeness, and Radiality.

### 2.6 Identification and Verification of the Biomarkers

The AKI-associated biomarkers in GSE61739 dataset were selected from candidate genes utilizing two machine learning methods. Firstly, the candidate genes were subjected to LASSO regression analysis using “glmnet” R package [22] to identify the feature genes when the lambda value was minimal. Meanwhile, SVM-RFE analysis was also conducted on the candidate genes using the “e1071” R package [23] to identify feature genes corresponding to the smallest Root Mean Square Error (RMSE). Subsequently, the hub genes of AKI were identified by intersecting the feature genes obtained from the two machine learning methods. Furthermore, the diagnostic performance of the hub genes was evaluated using Receiver Operating Characteristic (ROC) analysis, performed with the “multipleROC” R package [24]. Biomarkers of AKI diagnostic performance impact were finally screened based on the area under the ROC curve (AUC) value for each gene, where AUC ≥ 0.8 was usually considered as a model with good discriminatory power [25].

### 2.7 Construction of Tfs Regulatory Network

The potential TFs of the biomarkers in the kidney tissue were screened through the hTFtarget database (https://guolab.wchscu.cn/hTFtarget/#!/) [26]. The miRNAs targeting the biomarkers were predicted by the Encori database (https://rnasysu.com/encori/) to select miRNAs with experimental validation counts of ≥ 5. Thereafter, the TFs’ regulatory network was constructed using the Cytoscape3.8.0 software.

### 2.8 Prediction Of Targeted Drugs And Molecular Docking

The targeted drugs for the biomarkers were predicted using the “Enrichr” R package based on the Drug Signatures Database (DSigDB), aiming to identify bioactive compounds with potential regulatory effects in AKI [27]. Candidate drugs were first ranked according to their significance (*p*-values). To ensure clinical relevance and structural feasibility, the following filtering criteria was applied, (1) context-specific gene expression signatures involving individual cell lines (*e.g.*, “scriptaid MCF7 DOWN”) were excluded, (2) only compounds with available three-dimensional (3D) molecular structures in the PubChem database were retained, (3) compounds not suitable for therapeutic use in AKI, such as inorganic ions (*e.g.*, ZINC) or general oxidants (*e.g.*, hydrogen peroxide) were eliminated. After this multi-step filtering, the top-ranked remaining compounds were selected as drug candidates for molecular docking analysis.

The protein crystal structures of the biomarkers were retrieved from the Uniprot database (https://www.uniprot. org/), with the selection criteria prioritizing X-ray crystallography as the structure source. The structures based on the minimal resolution values (in Å) were selected for higher accuracy and well-defined positions, particularly when multiple options were available, to ensure shorter amino acid sequences. The 3D molecular structures of the candidate drugs were downloaded from the PubChem website (https://pubchem.ncbi.nlm.nih.gov/). If no ready-made 3D structures were available, small molecules were manually drawn or modified as needed. Before molecular docking, the protein and drug structures were processed using PyMOL software to remove water molecules, hydrogen atoms, and small, irrelevant molecules. Subsequently, ChemBioOffice software was applied to minimize the energy of the 3D structures of the drugs. Molecular docking was then conducted using AutoDockTools, with the receptor as the biomarker protein and the ligand as the drug molecule [28], using a binding energy threshold of <-5 kcal/mol for screening.

### 2.9 Statistical Analysis

The statistical analysis was conducted employing R software (version 4.3.0). Differences were analyzed using the Wilcoxon rank-sum test and Kruskal test. A *p*-value of <0.05 was considered statistically significant.

## 3 RESULTS

### 3.1 WGCNA Screened 498 Key Module Genes Associated with AKI

**Fig. (1):**
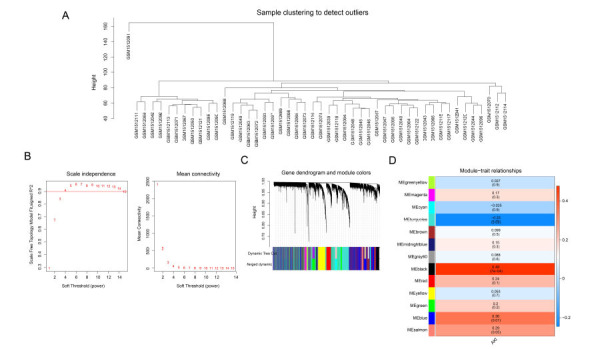
Screening of critical module genes associated with AKI through WGCNA. (**A**) Sample clustering tree to detect outliers in GSE61739 dataset; (**B**) Determining the optimal soft threshold (β) to ensure scale-free topology network; (**C**) Gene dendrogram and module colors; (**D**) Module-trait relationship heatmap between each module and AKI.

WGCNA was performed on GSE61739 dataset to screen critical module genes related to AKI. Clustering analysis revealed one outlier sample (Fig. **1A**), which was eliminated from the subsequent study. To establish a scale-free topology network, the optimal soft threshold was set to be 4 based on the intercept height (R^2^)>0.9 (Fig. **1B**). Thereafter, a total of 13 co-expression modules with a minimum gene number of 100 and merging height of 0.2 were acquired (Fig. **1C**). Module-trait relationship heatmap showed that the black module exhibited the strongest correlation with AKI (cor=0.48 and *p*=7e-04) (Fig. **1D**). After removing the unidentifiable genes in the black module, 498 critical module genes related to AKI were obtained for subsequent analysis.

### 3.2 Functional Enrichment Analysis on the 498 Key Module Genes

**Fig. (2):**
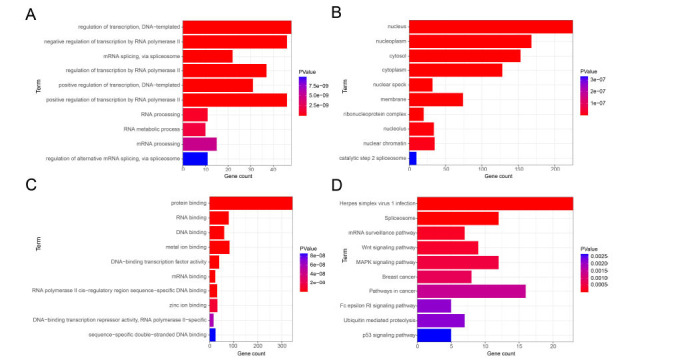
Functional enrichment analysis of the critical module genes. (**A**) GO enrichment in BP term; (**B**) GO enrichment in CC term; (**C**) GO enrichment in MF term; (**D**) Enriched KEGG pathways.

GO enrichment analysis revealed that in the BP category, the 498 key module genes were significantly enriched in biological processes such as regulation of transcription, RNA processing, RNA metabolic process, and mRNA processing (Fig. **2A**, Table **S1**), which are closely linked to inflammatory responses and cellular stress adaptation. In the CC category, genes were mainly localized in nucleus, nucleoplasm, cytosol, and cytoplasm (Fig. **2B**, Table **S1**), suggesting their involvement in intracellular signal transduction and immune gene regulation. In the MF category, these genes were primarily enriched in protein binding, RNA binding, DNA binding, and mRNA binding (Fig. **2C**, Table **S1**), potentially participating in transcriptional reprogramming during immune activation. KEGG pathway analysis further revealed that the 498 genes were notably involved in the spliceosome, mRNA surveillance, MAPK signaling pathway, Wnt signaling pathway, and p53 signaling pathway (Fig. **2D**, Table **S1**), several of which (*e.g.*, MAPK, Wnt, and p53) are widely implicated in immune regulation, apoptosis, and metabolic disturbance in AKI. These results suggested that the identified genes may participate in the immunometabolic dysregulation underlying AKI pathogenesis.

### 3.3 Screening of DEGs and Construction of PPI Network

**Fig. (3):**
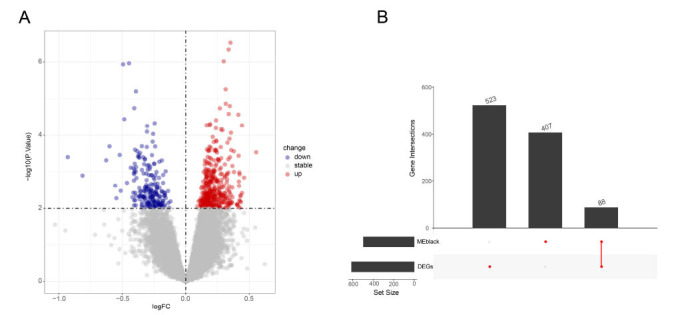
Recognition of differentially expressed genes (DEGs) related to AKI. (**A**) Volcano plot of DEGs between AKI samples and control samples in GSE61739 dataset; (**B**) Upset plot of DEGs and critical module genes.

Firstly, 611 DEGs between AKI samples and control samples in GSE61739 dataset were obtained (*p*<0.01) and visualized into a volcano plot (Fig. **3A**). Then, the 611 DEGs were intersected with 498 critical module genes to obtain 88 AKI-related DEGs for further study (Fig. **3B**).

**Fig. (4):**
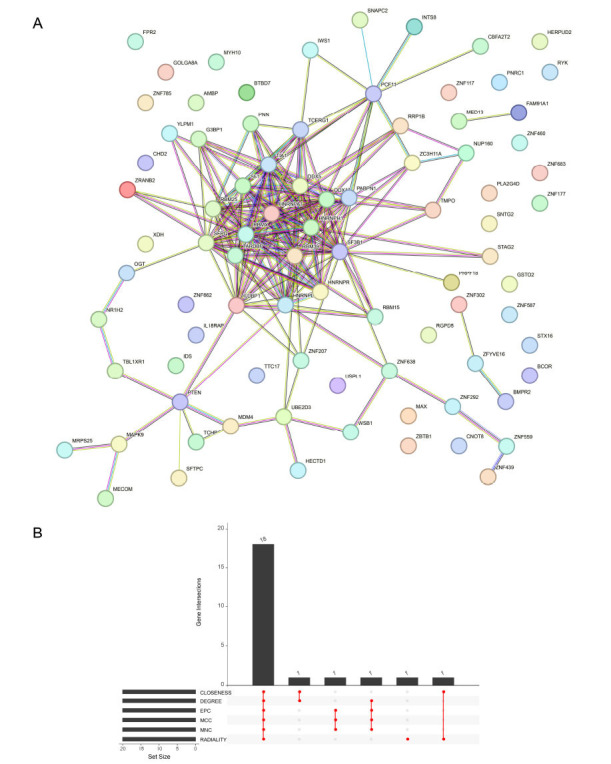
Screening of candidate genes related to AKI by PPI network analysis. (**A**) PPI network diagram of AKI-related DEGs; (**B**) Upset plot of top 20 important genes identified by 6 algorithms of cytoHubba plug-in.

The above 88 AKI-related DEGs were loaded into STRING database to develop a PPI network, which was then visualized by Cytoscape software (Fig. **4A**). Using six cytoHubba algorithms (MCC, MNC, Degree, EPC, Closeness, Radiality), 18 intersecting hub genes were identified as candidate genes for AKI (Fig. **4B**), forming the basis for subsequent biomarker screening and functional exploration.

### 3.4 Four Diagnostic Biomarkers of AKI Identified by Two Machine Learning Methods

**Fig. (5):**
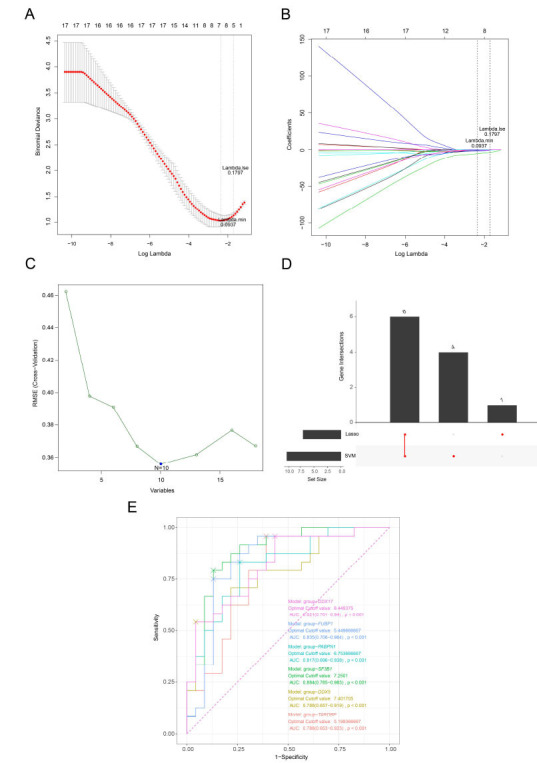
Identification and validation of diagnostic biomarkers of AKI. (**A**) Penalty parameter of LASSO regression analysis; (**B**) Coefficients of LASSO regression analysis; (**C**) Relationship of root-mean-square error (RMSE) and variables in SVM-RFE analysis; (**D**) Upset plot of feature genes of LASSO regression and SVM-RFE analysis; (**E**) ROC curve of 6 hub genes.

To screen biomarkers for AKI, LASSO regression analysis was first performed on the 18 candidate genes, yielding 7 feature genes at the optimal lambda value (lambda.min=0.0937) (Fig. **5A** and **B**). SVM-RFE analysis was also conducted on the 18 candidate genes, and 10 feature genes were obtained when the RMSE was minimal (Fig. **5C**). Next, 6 hub genes of AKI were acquired by intersecting the feature genes identified by the two algorithms (Fig. **5D**). According to ROC curve, it was found that the AUC value of the 6 hub genes (*DDX17*, *FUBP1*, *PABPN1*, *SF3B1*, *DDX5*, and *TARDBP*) was 0.821, 0.835, 0.817, 0.884, 0.788, and 0.788, respectively (Fig. **5E**). Notably, *DDX17*, *FUBP1*, *PABPN1* with AUC values exceeding 0.8, exhibited strong predictive performance and were therefore considered as potential diagnostic biomarkers for AKI, serving as a foundation for further experimental validation and translational research rather than immediate clinical applications.

### 3.5 Construction of Regulatory Networks and Molecular Docking Models for TFs of Biomarkers

The hTFtarget database was employed to predict the TFs for the 4 biomarkers, and 19 TFs for *SF3B1*, 10 TFs for *PABPN1*, 15 TFs for *FUBP1*, and 13 TFs for *DDX17* were obtained (Fig. **S1**). In addition, miRNAs with experimental validation counts of ≥ 5 targeting the 4 biomarkers were predicted using the Encori database, and 17 miRNAs targeting *SF3B1*, 58 miRNAs targeting *PABPN1*, 60 miRNAs targeting *FUBP1*, and 109 miRNAs targeting *DDX17* were identified (Fig. **S1**).

**Fig. (6):**
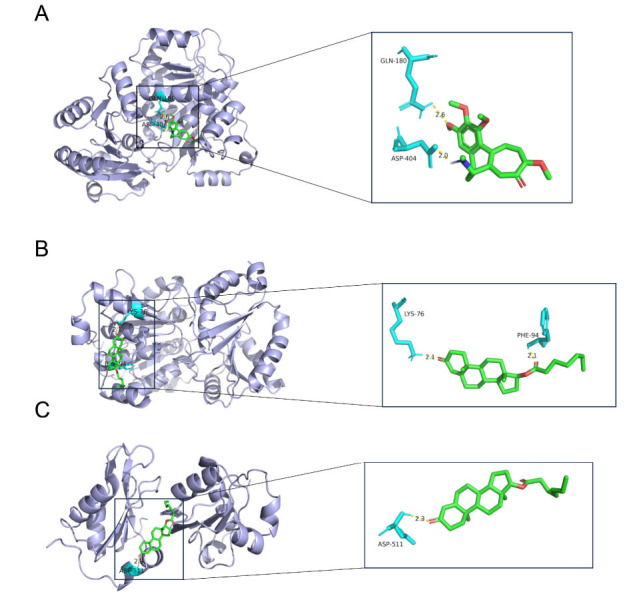
Prediction of targeted drugs and construction of molecular docking models. (**A**) Molecular docking result of *DDX17* and Demecolcine; (**B**) Molecular docking result of *DDX17* and Testosterone Enanthate; (**C**) Molecular docking result of *SF3B1* and Testosterone Enanthate.

Furthermore, two drugs (Demecolcine and Testosterone Enanthate) targeting the 4 biomarkers were predicted using the DSigDB database, and the 6uv3 protein of *DDX17*, 6y24 protein of *FUBP1*, 3ucg protein of *PABPN1*, and 4oz1 protein of *SF3B1* were acquired from the Uniprot database. Thereafter, 3 pairs of molecular docking models with binding energy <-5 kcal/mol were developed. The parameters of molecular docking are shown in Table **1**. Specifically, the binding energy of *DDX17* to Demecolcine was -7.46 kcal/mol, with the hydrogen bonding sites locating at GLN-180 and ASP-404 (Table **2** and Fig. **6A**), the binding energy of *DDX17* to Testosterone Enanthate was -7.5 kcal/mol, with the hydrogen bonding sites locating at LYS-76 and PHE-94 (Table **2** and Fig. **6B**), the binding energy of *SF3B1* to Testosterone Enanthate was -7.78 kcal/mol, with the hydrogen bonding site locating at ASP-511 (Table **2** and Fig. **6C**). In addition, the molecular docking residue parameters for the target and the drug are shown in Table **S2**.

## 4 DISCUSSION

AKI represents a prevalent clinical critical disease that is characterized by a rapid deterioration of renal function, and shows a poor prognosis and high mortality rate [29]. Current standard screening methods, for example, the detection of SCr levels, are not fully reliable for diagnosing early AKI [30]. Hence, it is of great significance to discover novel diagnostic biomarkers with a high specificity and sensitivity for AKI. In the present study, 498 critical module genes associated with AKI were identified using WGCNA. Subsequently, 4 diagnostic biomarkers with an AUC > 0.8 were selected using machine learning algorithms, demonstrating a strong predictive performance in AKI. Furthermore, several TFs and miRNAs targeting the 4 biomarkers were predicted. Two targeted drugs (Demecolcine and Testosterone Enanthate) were developed to construct molecular docking models. Overall, the results of this study provided a new perspective on early screening for AKI and its personalized treatment.

GO and KEGG enrichment analysis revealed that the 498 critical module genes associated with AKI were primarily involved in RNA processing, RNA metabolic process, RNA binding, Spliceosome, Wnt, MAPK, and p53 signaling pathways. RNA-binding proteins play essential roles in post-transcriptional regulation and protein synthesis, and also participate in the development of the ribosome and spliceosome [31]. Alternative splicing has been recently found to be linked to various renal diseases, and Lin *et al* verified the involvement of RNA-binding protein RBFOX1 in AKI [32]. In addition, Wnt signaling cascade is a highly conserved pathway that modulates stem cell differentiation, kidney development, and damage repair [33]. Increasing evidence has shown that the Wnt signaling pathway plays a critical role in controlling early nephrogenesis and is linked to the progression of multiple renal diseases [34], demonstrating its potential as a promising treatment target [35]. Mitogen-Activated Protein Kinase (MAPK) is a family of serine/threonine kinases that regulate cell proliferation, apoptosis, and inflammation [36]. After kidney damage, renal epithelial cells can stimulate a variety of signaling pathways, especially MAPK-relevant pathways, to facilitate the recovery of renal tubule structure and function [37]. Moreover, A study found that p53 participates in the pathogenesis of AKI and CKD through the crosstalk with Wnt signaling [38, 39]. These findings suggested that these pathways may be involved in AKI progression, thereby enhancing the understanding of its pathogenesis.

This study employed two machine learning algorithms to screen 4 biomarkers (AUC > 0.8) with significant diagnostic value in AKI, including *DDX17*, *FUBP1*, *PABPN1*, and *SF3B1*. DEAD-box RNA helicase 17 (*DDX17*) is an RNA-binding protein that exerts significant effects on DNA repair, histone modification, and miRNA regulation [40]. Previous research has reported that DDX17 is associated with the metastasis of clear-cell Renal Cell Carcinoma (ccRCC) [41]. *DDX17* exhibited a close clinical correlation with SCr level and glomerular filtration rate and has also been identified as one of the biomarkers for CKD progression [42]. Far Upstream element-Binding Protein 1 (*FUBP1*), an RNA- and DNA-binding protein, participates in both standard and pathological biological processes and is recognized as a cancerogenic factor in various malignancies [43]. Overexpression of *FUBP1* could promote tumor proliferation and oncogenesis in ccRCC [44]. Tu *et al*. indicated that FUBP1 can activate USP7 expression, which in turn promotes ccRcc progression by deubiquitinating and stabilizing HIF2α [45]. Besides, Zhou *et al* reported that circPTPN14 could exacerbate renal fibrosis *via* binding to *FUBP1*, thereby enhancing the transcription level of MYC [46] (a TF of *FUBP1* identified by our study). Poly(A) binding protein nuclear 1 (*PABPN1*), a universally expressed RNA-binding protein, plays a crucial role in modulating RNA processing [47]. Study showed that highly expressed *PABPN1* could facilitate the migration and invasion of ccRCC cells and is relevant to patients’ prognosis [48]. Splicing Factor 3b subunit 1 (*SF3B1*) is a major component of spliceosome that has been previously detected in Madin-Darby canine kidney cells. *SF3B1* also participates in RNA splicing and gene expression [49]. *SF3B1* mutation has been frequently detected in human malignant tumors and is associated with patient outcomes [50]. The aberrant expression of *SF3B1* could influence the transcript splicing in chromophobe renal cell carcinoma [51]. Although *DDX17*, *FUBP1*, *PABPN1,* and *SF3B1* have been reported in other renal diseases, this study is the first to associate them with the occurrence of AKI. All four proteins showed high diagnostic efficacy in AKI samples, suggesting that they may exhibit specific expression characteristics in acute injury states. In addition, combined with the functions of specific genes in RNA regulation, splicing, and stress response, these markers may play a crucial role in the early pathological process of AKI and have the potential to become diagnostic and therapeutic targets.

In addition, Demecolcine and Testosterone Enanthate are potential AKI-targeting agents screened based on the DSigDB database, and molecular docking results indicate strong binding ability to key markers such as *DDX17* and *SF3B1*. Demecolcine is a colchicine-based microtubule protein polymerization inhibitor, which is commonly used in mammals (*e.g.*, mice, cattle, sheep, and goats) to assist in oocyte removal [52], and its mechanism of intervention in cellular microtubule structure suggests that it may affect the dynamic stability of the cells involved in AKI. However, Demecolcine is mainly cleared by renal metabolism and has a narrow safety range, thus posing a potential risk of toxicity in patients with impaired renal function. Despite its good binding ability to AKI markers as shown by molecular docking analysis in this study, this drug is clinically valuable for patients with abnormal renal function, especially in the acute stage of injury. Testosterone Enanthate, on the other hand, is a common androgen replacement therapy drug widely used to regulate bone metabolism [53]. In combination with its binding ability to AKI-associated markers, this suggests that it may be involved in the molecular regulation of renal tissue injury through indirect mechanisms. Nevertheless, the inclusion of Demecolcine and Testosterone Enanthate as drug candidates in this study was mainly based on an initial screening from a structure-docking perspective, and subsequent systematic evaluation of their toxicological properties and dose-dependence in cellular and animal models is still needed to clarify the limits of their applicability and safety in acute kidney injury, to avoid potential therapeutic risks.

It is worth noting that the identified biomarkers may be influenced by potential confounding factors such as age, comorbidities, medication use, and the etiology of AKI, which were not fully controlled for in the public dataset. Additionally, genetic heterogeneity among AKI patients may lead to differential gene expression patterns, which could impact biomarker selection and interpretation. Therefore, while the four identified genes demonstrate promising diagnostic potential, their expression and relevance may vary across distinct AKI subtypes and patient populations. Future studies incorporating stratified analyses and multi-ethnic validation cohorts are warranted to clarify the specificity and generalizability of these biomarkers. Additionally, this study has some limitations that should be acknowledged. For example, it was modeled and validated based on a single dataset in the GEO database, which has a relatively small sample size and may be susceptible to platform bias and sample heterogeneity. Subsequent studies should integrate multiple public data platforms with multicenter clinical sample data to conduct cross-platform analysis and joint modeling to improve the robustness and generalization of the model. Secondly, current marker screening results have not been validated by independent cohorts or real clinical samples, limiting the evaluation of their clinical applicability. In this regard, a multi-method approach has been developed to validate key genes in prospective clinical cohorts and samples from diverse populations, assessing the consistency of their performance across different AKI subtypes. Thirdly, this study lacks *in vivo* and *in vitro* experiments to elucidate the mechanisms of the four markers in the development of AKI. Subsequently, cellular and animal models will be constructed to investigate the specific regulatory mechanisms of these markers in oxidative stress, inflammatory response, and apoptosis through gene overexpression/knockdown experiments. Fourthly, this study mainly relies on AUC values for diagnostic performance assessment and lacks a comprehensive evaluation of model robustness. Therefore, multiple validation strategies (*e.g.*, cross-validation, Bootstrap resampling, etc.) should be introduced to compare with more machine learning models and further optimize the marker screening process. Fifthly, although structural filtering and molecular docking were performed on the drug candidates, the lack of systematic assessment of their pharmacokinetic properties and potential toxicity risks may limit their clinical translation prospects. In the future, ADMET prediction tools or *in vivo* drug distribution experiments should be introduced to assess the pharmacological behavior and safety of drug candidates in the AKI state. Finally, the constructed TF-miRNA regulatory network is based on database prediction and computational modeling inference, and lacks experimental evidence to support its real biological regulatory relationship. Subsequently, the key regulatory axes will be validated using molecular experimental techniques, such as the dual luciferase reporter gene system and RIP-qPCR, to enhance the credibility and explanatory power of the network construction.

## CONCLUSION

In this study, four AKI markers (*DDX17*, *FUBP1*, *PABPN1*, and *SF3B1*) with potential diagnostic value were screened based on WGCNA with dual machine learning algorithms demonstrating good prediction performance. Demecolcine and Testosterone Enanthate were further predicted and screened as potential target drugs, and their binding activities with the markers were verified by molecular docking. The findings provided a theoretical basis for early diagnosis and targeted intervention in AKI. In the future, functional experiments will be conducted to investigate the expression characteristics and mechanistic roles of the markers in different AKI subtypes, and their applicability will be validated in combination with clinical samples, thereby promoting the potential applications of these biomarkers and the redevelopment of drugs.

## Figures and Tables

**Table 1:** Parameters in molecular docking.

Term	Spacing	npts	Center
DDX17_ Demecolcine	0.592	126 126 126	49.453 -16.206 -34.034
DDX17_ Testosterone Enanthate	0.581	126 36 126	49.453 -16.206 -34.034
SF3B1_ Testosterone Enanthate	0.481	126 126 102	16.631 -53.032 98.943

**Table 2:** Binding energy of molecular docking.

Compound CID	Molecula_name	Gene_name	PDB_ID	Energy (kcal/mol)
220401	Demecolcine	DDX17	6uv3	-7.46
9416	Testosterone Enanthate	DDX17	6uv3	-7.5
9416	Testosterone Enanthate	SF3B1	4oz1	-7.78

## Data Availability

The datasets generated and/or analyzed during the current study are available in the [GSE61739] repository, (https://www.ncbi.nlm.nih.gov/geo/query/acc.cgi?accGSE61 739).

## LIST OF ABBREVIATIONS

AKI: Acute Kidney Injury
AUC: Area Under ROC Curve
BP: Biological Process
Cc: Cellular Component
ccRCC: clear-cell Renal-Cell Carcinoma
CKD: Chronic Kidney Disease
DDX17: DEAD-box RNA helicase 17
DEGs: Differentially Expressed Genes
DSigDB: Drug Signatures Database
EPC: Edge Percolated Component
FUBP1: Far Upstream element-Binding Protein 1
Geo: Gene Expression Omnibus
Go: Gene Ontology
KDIGO: Kidney Disease Improving Global Outcomes
KEGG: Kyoto Encyclopedia of Genes and Genomes
LASSO: Least Absolute Shrinkage and Selection Operator
MCC: Maximal Clique Centrality
MAPK: Mitogen-Activated Protein Kinase
Mf: Molecular Function
MNC: Maximum Neighborhood Component
PABPN1: Poly(A) Binding Protein Nuclear 1
PPI: Protein-Protein Interaction
Rmse: Root-Mean-Square Error
Roc: Receiver Operating Characteristic
Scr: Serum Creatinine
SF3B1: Splicing Factor 3b subunit 1
STRING: Search Tool for the Retrieval of Interacting Genes
SVM-RFE: Support Vector Machine-Recursive Feature Elimination
TFS: Transcription Factors
WGCNA: Weighted Gene Co-Expression Network Analysis
