# Supplementary material for: Diagnostic Biomarkers and Targeted Drug Prediction for Acute Kidney Injury: A Computational Approach
Source: Endocr Metab Immune Disord Drug Targets. 2025 Jul 30;26:E18715303417142. doi: 10.2174/0118715303417142250724042300 (PMC13334258; doi:10.2174/0118715303417142250724042300)
Supplement: Supplementary file 1 [file EMIDDT-26-E18715303417142_SD1.pdf]

# Supplementary Material

## Diagnostic Biomarkers and Targeted Drug Prediction for Acute Kidney Injury: A Computational Approach

Liuyin Zhou[1,#], Lian Pan[2,#], Jiayang Gao[3,#], Yi Jiang[4], Tingting Li[4,*] and Ruoqing Li[4,*]

[1]*Department of Respiratory Medicine, Chongqing University Central Hospital, Chongqing Emergency Medical Center, Chongqing Key Laboratory of Emergency Medicine, Chongqing, 400014, China;* [2]*Department of Plastic Surgery, Chongqing University Central Hospital, Chongqing Emergency Medical Center, Chongqing Key Laboratory of Emergency Medicine, Chongqing, 400014, China;* [3]*Department of Biopharmaceutical, College of Food Science and Technology, Shanghai Ocean University, Shanghai, 200090, China;* [4]*Department of General Medicine, Chongqing University Central Hospital, Chongqing Emergency Medical Center, Chongqing Key Laboratory of Emergency Medicine, Chongqing, 400014, China*

**Table S1. Results of GO and KEGG enrichment analysis.**

| #Term | Database | ID | Input number | Background number | P-Value | Corrected P-Value | Input | Hyperlink |
|---|---|---|---|---|---|---|---|---|
| regulation of transcription, DNA-templated | Gene Ontology | GO:0006355 | 48 | 773 | 7.23E-19 | 2.66E-16 | ZNF217\|ZNF195\|ZNF23\|PHF12\|MED23\|TMPO\|NFYB\|NFYA\|ZNF460\|ZNF559\|ZNF3\|ZNF570\|ZBTB38\|CNOT8\|ZNF785\|JMJD1C\|ZNF578\|RYBP\|ZNF662\|ZNF439\|DMTF1\|ZNF281\|ZNF207\|ZNF41\|ZBTB4\|ZNF302\|ZNF84\|NR2C2\|ZNF587\|ZNF586\|SNAI3\|RNF20\|TARDBP\|ZNF675\|RPS6KA4\|ZNF616\|ZNF619\|HNRNPD\|ZNF717\|ZNF862\|HOXD13\|ZNF138\|NFX1\|ZNF33A\|ZNF117\|ACTR5\|ZNF502\|EWSR1 | http://amigo.geneontology.org/amigo/term/GO:0006355 |
| negative regulation of transcription by RNA polymerase II | Gene Ontology | GO:0000122 | 46 | 832 | 2.60E-16 | 6.08E-14 | BPTF\|ZNF217\|BCOR\|PHF12\|SFTPC\|ZNF692\|TCERG1\|UBE2D3\|SUDS3\|N4BP2L2\|DUSP26\|DLG1\|NFX1\|SUPT5H\|CGGBP1\|MEF2A\|CBFA2T2\|KLF3\|TBX15\|ZNF345\|RYBP\|NR1H2\|ZNF281\|ZBTB1\|CDX2\|ZBTB4\|HEY2\|NR2C2\|PPARD\|NR2C1\|SATB1\|SHH\|SNAI3\|MDM4\|ZNF675\|TBL1XR1\|ETV7\|MAX\|ZNF177\|PSMD10\|EPC1\|DDX5\|PURA\|GATAD2B\|CTBP2\|KDM5A | http://amigo.geneontology.org/amigo/term/GO:0000122 |
| mRNA splicing, via spliceosome | Gene Ontology | GO:0000398 | 22 | 246 | 3.86E-12 | 6.22E-10 | RBM6\|SF3B1\|CDC5L\|RBMXL2\|HNRNPH1\|HNRNPH3\|HNRNPR\|PCF11\|HNRNPL\|RBM15\|HNRNPD\|TRA2B\|PABPN1\|HNRNPA3\|HNRNPA1\|SNRPN\|METT | http://amigo.geneontology.org/amigo/term/GO:0000398 |

| | | | | | | | | |
|---|---|---|---|---|---|---|---|---|
| | | | | | | | L3\|CSTF3\|RBMX\|SPEN\|DDX5\|PNN | |
| regulation of transcription by RNA polymerase II | Gene Ontology | GO:0006357 | 37 | 751 | 6.17E-12 | 9.35E-10 | BPTF\|FOXP1\|FOXR2\|CDX2\|DMTF1\|RCOR3\|JMJD1C\|DDX17\|SUPT5H\|KDM4C\|MED13\|NFE2\|PHIP\|DLX5\|BRWD1\|MED23\|BRCA1\|ZBTB1\|MED17\|NR2C2\|PPARD\|NR2C1\|SATB1\|BRWD3\|MDM4\|TBL1XR1\|ETV7\|MAX\|RFX7\|EPC2\|EPC1\|DDX5\|TIAL1\|OGT\|KDM3A\|TFDP1\|ZNF292 | http://amigo.geneontology.org/amigo/term/GO:0006357 |
| positive regulation of transcription, DNA-templated | Gene Ontology | GO:0045893 | 31 | 556 | 1.83E-11 | 2.62E-09 | FBXW11\|CDKN1C\|CDX2\|NFYB\|NFYA\|ZXDC\|TAF15\|MECOM\|MED13\|NFE2\|PHIP\|DYRK1A\|RYBP\|HNRNPD\|NR1H2\|ZNF281\|BRCA1\|MED17\|ANKRD49\|PPARD\|TP53INP1\|DLX5\|SHH\|HEY2\|RNF20\|NAA15\|TBL1XR1\|TP73\|EPC1\|KDM3A\|KDM5A | http://amigo.geneontology.org/amigo/term/GO:0045893 |
| positive regulation of transcription by RNA polymerase II | Gene Ontology | GO:0045944 | 46 | 1159 | 1.93E-11 | 2.62E-09 | BPTF\|LAGE3\|SFTPC\|RRP1B\|TCERG1\|CDC5L\|NFYB\|IL1A\|RBMX\|LRP5L\|DDX17\|MAML2\|ASXL1\|SUPT5H\|SIX2\|ZBTB38\|MED13\|KLF3\|PPP1R12A\|PHIP\|DLX5\|GTF2I\|NR1H2\|TBX15\|BRCA1\|FOXJ2\|MED17\|NR2C2\|PPARD\|MYSM1\|NFATC2IP\|SHH\|HEY2\|RPS6KA4\|TBL1XR1\|MAX\|HOXD13\|TP73\|EPC1\|MEF2A\|BMPR2\|OGT\|CTBP2\|KDM3A\|TFDP1\|ZNF292 | http://amigo.geneontology.org/amigo/term/GO:0045944 |
| RNA processing | Gene Ontology | GO:0006396 | 11 | 58 | 8.78E-10 | 1.08E-07 | RBM6\|PABPN1\|DDX17\|RBM39\|DDX54\|HNRNPH1\|HNRNPH3\|HNRNPL\|NOL9\|HNRNPD\|DUSP11 | http://amigo.geneontology.org/amigo/term/GO:0006396 |
| RNA metabolic process | Gene Ontology | GO:0016070 | 10 | 46 | 1.62E-09 | 1.90E-07 | METTL3\|HNRNPD\|HNRNPA3\|HNRNPR\|HNRNPA1\|DDX54\|RBMX\|HNRNPH1\|HNRNPL\|DUSP11 | http://amigo.geneontology.org/amigo/term/GO:0016070 |
| mRNA processing | Gene Ontology | GO:0006397 | 15 | 158 | 4.96E-09 | 5.55E-07 | RBM26\|ZRANB2\|RRP1B\|TCERG1\|HNRNPR\|METTL3\|SF3B1\|IWS1\|RBM25\|TSEN2\|PRPF18\|CPSF6\|RBM39\|PRPF38B\|TARDBP | http://amigo.geneontology.org/amigo/term/GO:0006397 |
| regulation of alternative mRNA splicing, via spliceosome | Gene Ontology | GO:0000381 | 11 | 75 | 9.75E-09 | 1.01E-06 | DDX17\|HNRNPA1\|RBMX\|SPEN\|DDX5\|RBM25\|HNRNPL\|RBM15\|DYRK1A\|RBM19\|TRA2B | http://amigo.geneontology.org/amigo/term/GO:0000381 |
| RNA splicing | Gene Ontology | GO:0008380 | 14 | 160 | 4.16E-08 | 3.83E-06 | ZNF638\|TAF15\|ZRANB2\|RRP1B\|TCERG1\|PRPF38B\|SF3B1\|IWS1\|RBM25\|SNRPN\|HNRNPH3\|PRPF18\|RBM39\|TARDBP | http://amigo.geneontology.org/amigo/term/GO:0008380 |
| viral process | Gene Ontology | GO:0016032 | 23 | 456 | 4.38E-08 | 3.89E-06 | FBXW11\|SATB1\|RRP1B\|SF3B1\|DLG1\|NFX1\|MAML2\|AMBP\|RBM15\|DYRK1A\|ZC3H7B\|ZNF683\|NUP160\|HNRNPA1\|EIF4H\|WRAP53\|TAPT1\|TAF4\|OGT\|TP73\|SPEN\|G3BP1\|RNF8 | http://amigo.geneontology.org/amigo/term/GO:0016032 |
| negative regulation of transcription, DNA- | Gene Ontology | GO:0045892 | 25 | 536 | 4.83E-08 | 4.15E-06 | ZNF217\|FBXW11\|PHF12\|CDKN1C\|SUDS3\|RCOR3\|FOXP1\|MECOM\|SIX2\|CBFA2T2\|ZBTB38\|RBM15\|NR1H2\|ZNF281\|BR | http://amigo.geneontology.org/amigo/term/GO:0045892 |

| | | | | | | | | |
|---|---|---|---|---|---|---|---|---|
| templated | | | | | | | CA1\|PHC3\|ZBTB4\|PPARD\|BCOR\|HEY2\|EPC1\|SPEN\|MBD1\|ZNF91\|CTBP2 | |
| regulation of RNA splicing | Gene Ontology | GO:0043484 | 8 | 43 | 2.05E-07 | 1.56E-05 | PTBP2\|RRP1B\|HNRNPH1\|HNRNPH3\|HNRNPL\|CLK1\|CLK2\|TRA2B | http://amigo.geneontology.org/amigo/term/GO:0043484 |
| protein deubiquitination | Gene Ontology | GO:0016579 | 16 | 262 | 4.71E-07 | 3.19E-05 | BRCA1\|ZRANB1\|USP48\|ASXL1\|USP34\|USP54\|OTUD3\|PSMD10\|SUDS3\|PTEN\|MYSM1\|OGT\|ACTR5\|MDM4\|INO80D\|USP36 | http://amigo.geneontology.org/amigo/term/GO:0016579 |
| apoptotic process | Gene Ontology | GO:0006915 | 23 | 546 | 9.02E-07 | 5.67E-05 | BIRC7\|TCHP\|UBE2D3\|SUDS3\|IL1A\|MECOM\|RPS6KB1\|RRP1B\|RYBP\|BRCA1\|TRAF7\|PPARD\|TP53INP1\|PHLPP1\|BNIP2\|OGT\|PSMD10\|TIA1\|PTEN\|MEF2A\|MBD1\|TIAL1\|GZMM | http://amigo.geneontology.org/amigo/term/GO:0006915 |
| transcription by RNA polymerase II | Gene Ontology | GO:0006366 | 13 | 187 | 1.46E-06 | 8.72E-05 | NFX1\|TAF4\|TAF15\|ETV7\|SUPT5H\|RBMX\|IWS1\|SNAPC2\|NFYA\|MBD1\|ZNF345\|TFDP1\|TARDBP | http://amigo.geneontology.org/amigo/term/GO:0006366 |
| heart development | Gene Ontology | GO:0007507 | 12 | 189 | 8.77E-06 | 0.000471 | BCOR\|SRI\|KCNAB1\|PPARD\|PTEN\|MEF2A\|MBD1\|NF1\|PHIP\|SHH\|EDNRA\|CAD | http://amigo.geneontology.org/amigo/term/GO:0007507 |
| transcription initiation from RNA polymerase II promoter | Gene Ontology | GO:0006367 | 11 | 160 | 1.03E-05 | 0.00054 | NR2C1\|TAF4\|MAML2\|MED23\|NR2C2\|PTEN\|PPARD\|MED13\|TAF15\|MED17\|NR1H2 | http://amigo.geneontology.org/amigo/term/GO:0006367 |
| regulation of gene expression | Gene Ontology | GO:0010468 | 13 | 235 | 1.51E-05 | 0.000779 | ATXN7L3B\|NF1\|MECOM\|ZNF683\|CGGBP1\|FUBP1\|TP73\|POGZ\|FOXP1\|ZNF345\|SHH\|MAPK9\|POFUT2 | http://amigo.geneontology.org/amigo/term/GO:0010468 |
| negative regulation of G0 to G1 transition | Gene Ontology | GO:0070317 | 6 | 39 | 1.87E-05 | 0.000945 | BRCA1\|MAX\|EPC1\|PHC3\|RYBP\|TFDP1 | http://amigo.geneontology.org/amigo/term/GO:0070317 |
| cellular response to DNA damage stimulus | Gene Ontology | GO:0006974 | 13 | 248 | 2.58E-05 | 0.00128 | BRCA1\|ZBTB1\|METTL3\|ZBTB4\|VAV3\|RNF169\|TLK2\|TP73\|FMN2\|ZBTB38\|FOXP1\|RNF8\|CDKN2AIP | http://amigo.geneontology.org/amigo/term/GO:0006974 |
| dosage compensation by inactivation of X chromosome | Gene Ontology | GO:0009048 | 4 | 11 | 2.82E-05 | 0.001372 | SPEN\|BRCA1\|METTL3\|RBM15 | http://amigo.geneontology.org/amigo/term/GO:0009048 |
| response to insulin | Gene Ontology | GO:0032868 | 7 | 65 | 3.11E-05 | 0.001482 | SESN3\|MAX\|VGF\|MTOR\|EPM2AIP1\|OGT\|CAD | http://amigo.geneontology.org/amigo/term/GO:0032868 |
| rhythmic process | Gene Ontology | GO:0048511 | 7 | 66 | 3.40E-05 | 0.001592 | FBXW11\|MAML2\|DDX5\|NFYA\|MTOR\|MAPK9\|TARDBP | http://amigo.geneontology.org/amigo/term/GO:0048511 |
| branching involved in blood vessel morphogenesis | Gene Ontology | GO:0001569 | 5 | 29 | 5.84E-05 | 0.002552 | SPEN\|SHH\|FGF8\|RBM15\|EDNRA | http://amigo.geneontology.org/amigo/term/GO:0001569 |
| protein ubiquitination | Gene Ontology | GO:0016567 | 18 | 486 | 6.87E-05 | 0.002765 | ASB10\|NFX1\|FBXW11\|ARIH2\|TRAF7\|BIRC7\|TRIM32\|RNF169\|UBE2D3\|BRCA1\|HERC1\|PCNP\|DCAF17\|NSMCE1\|RNF8\| | http://amigo.geneontology.org/amigo/term/GO:0016567 |

| | | | | | | | | |
|---|---|---|---|---|---|---|---|---|
| | | | | | | | RNF26\|RNF20\|CNOT4 | |
| nuclear migration | Gene Ontology | GO:0007097 | 4 | 15 | 7.70E-05 | 0.003055 | MYH10\|SYNE2\|FBXW11\|CLMN | http://amigo.geneontology.org/amigo/term/GO:0007097 |
| positive regulation of retinoic acid receptor signaling pathway | Gene Ontology | GO:0048386 | 3 | 5 | 0.0001 | 0.003692 | ASXL1\|CTBP2\|NR2C1 | http://amigo.geneontology.org/amigo/term/GO:0048386 |
| myoblast differentiation | Gene Ontology | GO:0045445 | 4 | 18 | 0.000141 | 0.004851 | SHH\|DDX5\|KCNAB1\|DDX17 | http://amigo.geneontology.org/amigo/term/GO:0045445 |
| regulation of cell shape | Gene Ontology | GO:0008360 | 9 | 150 | 0.000173 | 0.005869 | DLG1\|MYH10\|ARHGEF18\|MKLN1\|PHIP\|BRWD3\|BRWD1\|RASA1\|ARHGAP18 | http://amigo.geneontology.org/amigo/term/GO:0008360 |
| positive regulation of proteolysis | Gene Ontology | GO:0045862 | 4 | 20 | 0.000201 | 0.006731 | TRIM32\|FBXW11\|CLN6\|OGT | http://amigo.geneontology.org/amigo/term/GO:0045862 |
| forebrain morphogenesis | Gene Ontology | GO:0048853 | 3 | 8 | 0.000287 | 0.009258 | PTEN\|NF1\|FGF8 | http://amigo.geneontology.org/amigo/term/GO:0048853 |
| positive regulation of translation | Gene Ontology | GO:0045727 | 6 | 73 | 0.000448 | 0.013584 | METTL3\|RPS6KB1\|UPF3A\|MTOR\|HNRNPL\|HNRNPD | http://amigo.geneontology.org/amigo/term/GO:0045727 |
| regulation of transcription by RNA polymerase III | Gene Ontology | GO:0006359 | 3 | 10 | 0.000489 | 0.014655 | BRCA1\|POLR3C\|ZNF345 | http://amigo.geneontology.org/amigo/term/GO:0006359 |
| cell population proliferation | Gene Ontology | GO:0008283 | 8 | 142 | 0.000585 | 0.016758 | MYH10\|USPL1\|IL18RAP\|DLX5\|SIX2\|PPARD\|FAM83B\|EDNRA | http://amigo.geneontology.org/amigo/term/GO:0008283 |
| fibroblast growth factor receptor signaling pathway | Gene Ontology | GO:0008543 | 6 | 80 | 0.000705 | 0.019325 | FAT4\|HNRNPA1\|TIA1\|HNRNPH1\|TIAL1\|FGF8 | http://amigo.geneontology.org/amigo/term/GO:0008543 |
| positive regulation of double-strand break repair | Gene Ontology | GO:2000781 | 3 | 12 | 0.000764 | 0.020295 | SPIRE2\|WRAP53\|FMN2 | http://amigo.geneontology.org/amigo/term/GO:2000781 |
| regulation of proteolysis | Gene Ontology | GO:0030162 | 3 | 12 | 0.000764 | 0.020295 | SHH\|C2CD3\|SPOP | http://amigo.geneontology.org/amigo/term/GO:0030162 |
| protein dephosphorylation | Gene Ontology | GO:0006470 | 8 | 149 | 0.000789 | 0.020752 | DLG1\|PTPRN2\|FBXW11\|PTEN\|PPP1R12A\|PHLPP1\|DUSP11\|DUSP26 | http://amigo.geneontology.org/amigo/term/GO:0006470 |
| neuron projection development | Gene Ontology | GO:0031175 | 7 | 116 | 0.000874 | 0.02275 | RAB35\|EFHD1\|RYK\|CLMN\|HERC1\|TMEM108\|NCDN | http://amigo.geneontology.org/amigo/term/GO:0031175 |
| negative regulation of | Gene Ontol- | GO:2000678 | 3 | 13 | 0.000932 | 0.02377 | ZNF675\|SRI\|HEY2 | http://amigo.geneontolo- |

| | | | | | | | | |
|---|---|---|---|---|---|---|---|---|
| transcription regulatory region DNA binding | ogy | | | | | | | gy.org/amigo/term/GO:2000678 |
| negative regulation of transcription elongation from RNA polymerase II promoter | Gene Ontology | GO:0034244 | 3 | 13 | 0.000932 | 0.02377 | SHH\|TCERG1\|RNF8 | http://amigo.geneontology.org/amigo/term/GO:0034244 |
| negative regulation of proteasomal ubiquitin-dependent protein catabolic process | Gene Ontology | GO:0032435 | 4 | 32 | 0.000992 | 0.025058 | SHH\|RYBP\|OGT\|TLK2 | http://amigo.geneontology.org/amigo/term/GO:0032435 |
| transcription, DNA-templated | Gene Ontology | GO:0006351 | 6 | 87 | 0.001063 | 0.026599 | ASXL1\|EPC1\|MEF2A\|CNOT8\|POLR3C\|CBFA2T2 | http://amigo.geneontology.org/amigo/term/GO:0006351 |
| smooth muscle tissue development | Gene Ontology | GO:0048745 | 3 | 14 | 0.001121 | 0.026746 | SHH\|DLG1\|NF1 | http://amigo.geneontology.org/amigo/term/GO:0048745 |
| ventricular septum morphogenesis | Gene Ontology | GO:0060412 | 4 | 34 | 0.001219 | 0.028818 | SPEN\|BMPR2\|HEY2\|RBM15 | http://amigo.geneontology.org/amigo/term/GO:0060412 |
| oxidation-reduction process | Gene Ontology | GO:0055114 | 16 | 525 | 0.001303 | 0.030191 | GSTO2\|KDM3A\|SESN3\|CRYZL1\|ALDH3A1\|KCNAB1\|SCD5\|KDM4C\|TMX4\|RRM2B\|JMJD1C\|KDM2A\|RSBN1\|CTBP2\|VKORC1L1\|KDM5A | http://amigo.geneontology.org/amigo/term/GO:0055114 |
| histone H3-K9 demethylation | Gene Ontology | GO:0033169 | 3 | 15 | 0.001333 | 0.030191 | KDM4C\|KDM3A\|JMJD1C | http://amigo.geneontology.org/amigo/term/GO:0033169 |
| negative regulation of macroautophagy | Gene Ontology | GO:0016242 | 3 | 15 | 0.001333 | 0.030191 | POGZ\|MTOR\|USP36 | http://amigo.geneontology.org/amigo/term/GO:0016242 |
| signal transduction | Gene Ontology | GO:0007165 | 25 | 1013 | 0.001336 | 0.030191 | RYK\|ZFYVE16\|TNFSF18\|FAM83B\|EDNRA\|FAM13A\|RPS6KB1\|CDKL2\|PPP1R12A\|MKLN1\|COPA\|VGF\|SRI\|NCR1\|PEX11A\|APPL1\|PHLPP1\|RASA1\|SCUBE3\|INPP1\|CSNK1A1\|OGT\|ENHO\|VAV2\|PITPNC1 | http://amigo.geneontology.org/amigo/term/GO:0007165 |
| ubiquitin-dependent protein catabolic process | Gene Ontology | GO:0006511 | 11 | 292 | 0.001511 | 0.033861 | USP48\|ARIH2\|SPOP\|TRIM32\|UBE2D3\|RNF8\|ANAPC1\|USP34\|RNF20\|USP36\|CNOT4 | http://amigo.geneontology.org/amigo/term/GO:0006511 |
| small GTPase mediated signal transduction | Gene Ontology | GO:0007264 | 6 | 94 | 0.001547 | 0.033961 | RGR\|SOS2\|ARHGEF18\|VAV3\|VAV2\|ARHGAP18 | http://amigo.geneontology.org/amigo/term/GO:0007264 |
| regulation of protein kinase B signaling | Gene Ontology | GO:0051896 | 3 | 16 | 0.001568 | 0.033961 | PTEN\|GRM2\|SESN3 | http://amigo.geneontology.org/amigo/term/GO:0051896 |
| inositol | Gene | GO:00 | 3 | 16 | 0.001568 | 0.033961 | PTEN\|INPP1\|INPP5A | http://amigo.gen |

| | | | | | | | | |
|---|---|---|---|---|---|---|---|---|
| phosphate dephosphorylation | Ontology | 46855 | | | | | | eontology.org/amigo/term/GO:0046855 |
| anterior/posterior axis specification | Gene Ontology | GO:0009948 | 3 | 16 | 0.001568 | 0.033961 | CDX2\|HEY2\|SIX2 | http://amigo.geneontology.org/amigo/term/GO:0009948 |
| negative regulation of apoptotic process | Gene Ontology | GO:0043066 | 15 | 487 | 0.00165 | 0.03544 | NAA15\|BAG4\|BIRC7\|TNFSF18\|RPS6KB1\|PSMD10\|FMN2\|PTEN\|PPARD\|MBD1\|PHIP\|SHH\|MDM4\|RASA1\|BNIP2 | http://amigo.geneontology.org/amigo/term/GO:0043066 |
| anterior/posterior pattern specification | Gene Ontology | GO:0009952 | 6 | 96 | 0.001712 | 0.036455 | BPTF\|LRP5L\|HOXD13\|BMPR2\|VANGL2\|HEY2 | http://amigo.geneontology.org/amigo/term/GO:0009952 |
| regulation of circadian rhythm | Gene Ontology | GO:0042752 | 5 | 65 | 0.001766 | 0.037312 | KDM2A\|MTOR\|MAPK9\|HNRNPD\|TARDBP | http://amigo.geneontology.org/amigo/term/GO:0042752 |
| embryo development | Gene Ontology | GO:0009790 | 3 | 17 | 0.001828 | 0.038302 | DLG1\|DLX5\|CDX2 | http://amigo.geneontology.org/amigo/term/GO:0009790 |
| cell differentiation | Gene Ontology | GO:0030154 | 17 | 596 | 0.001846 | 0.038357 | NAA15\|ZNF3\|ETV7\|MECOM\|CDX2\|ANKRD49\|MEF2A\|KRTDAP\|NR2C2\|PURA\|NR2C1\|TSPY1\|FGF8\|DLX5\|PPARD\|HEY2\|NR1H2 | http://amigo.geneontology.org/amigo/term/GO:0030154 |
| heart morphogenesis | Gene Ontology | GO:0003007 | 4 | 39 | 0.001941 | 0.039393 | ASXL1\|FGF8\|MTOR\|FAT4 | http://amigo.geneontology.org/amigo/term/GO:0003007 |
| positive regulation of transcription of Notch receptor target | Gene Ontology | GO:0007221 | 3 | 18 | 0.002113 | 0.042166 | SPEN\|MAML2\|RBM15 | http://amigo.geneontology.org/amigo/term/GO:0007221 |
| response to nutrient | Gene Ontology | GO:0007584 | 5 | 68 | 0.002127 | 0.042166 | PTEN\|ALDH3A1\|MTOR\|TBXA2R\|OGT | http://amigo.geneontology.org/amigo/term/GO:0007584 |
| protein autoubiquitination | Gene Ontology | GO:0051865 | 5 | 69 | 0.002258 | 0.044428 | BRCA1\|NFX1\|UBE2D3\|RNF8\|CNOT4 | http://amigo.geneontology.org/amigo/term/GO:0051865 |
| chromatin remodeling | Gene Ontology | GO:0006338 | 6 | 102 | 0.002287 | 0.044646 | BPTF\|ZBTB1\|SATB1\|KDM4C\|MYSM1\|KDM5A | http://amigo.geneontology.org/amigo/term/GO:0006338 |
| intracellular transport | Gene Ontology | GO:0046907 | 3 | 19 | 0.002425 | 0.044955 | SPIRE2\|STBD1\|FMN2 | http://amigo.geneontology.org/amigo/term/GO:0046907 |
| androgen receptor signaling pathway | Gene Ontology | GO:0030521 | 3 | 19 | 0.002425 | 0.044955 | DDX5\|KDM3A\|DDX17 | http://amigo.geneontology.org/amigo/term/GO:0030521 |
| male genitalia development | Gene Ontology | GO:0030539 | 3 | 19 | 0.002425 | 0.044955 | SHH\|HOXD13\|FGF8 | http://amigo.geneontology.org/amigo/term/GO:0030539 |
| RNA methylation | Gene Ontology | GO:0001510 | 3 | 19 | 0.002425 | 0.044955 | SPEN\|METTL3\|RBM15 | http://amigo.geneontology.org/amigo/te |

| | | | | | | | | |
|---|---|---|---|---|---|---|---|---|
| | | | | | | | | rm/GO:0001510 |
| branching involved in ureteric bud morphogenesis | Gene Ontology | GO:0001658 | 4 | 42 | 0.002493 | 0.045573 | SHH\|DLG1\|FGF8\|FAT4 | http://amigo.geneontology.org/amigo/term/GO:0001658 |
| mRNA transport | Gene Ontology | GO:0051028 | 4 | 42 | 0.002493 | 0.045573 | IWS1\|UPF3A\|HNRNPA1\|EIF5A | http://amigo.geneontology.org/amigo/term/GO:0051028 |
| protein polyubiquitination | Gene Ontology | GO:0000209 | 10 | 267 | 0.002537 | 0.045751 | HECTD2\|FBXW11\|ARIH2\|SPOP\|TRIM32\|PSMD10\|UBE2D3\|MKRN1\|WSB1\|RNF20 | http://amigo.geneontology.org/amigo/term/GO:0000209 |
| regulation of GTPase activity | Gene Ontology | GO:0043087 | 5 | 71 | 0.002539 | 0.045751 | VAV3\|VAV2\|MTOR\|NF1\|RAB3GAP1 | http://amigo.geneontology.org/amigo/term/GO:0043087 |
| DNA repair | Gene Ontology | GO:0006281 | 9 | 224 | 0.002621 | 0.046489 | CDC5L\|ZBTB1\|EPC2\|PDS5A\|UBE2D3\|MUS81\|RRM2B\|WRAP53\|INO80D | http://amigo.geneontology.org/amigo/term/GO:0006281 |
| multicellular organism development | Gene Ontology | GO:0007275 | 14 | 462 | 0.00268 | 0.046489 | ARIH2\|ZMYM4\|DKK4\|HOXD13\|FMN2\|KLF3\|NFE2\|PHC3\|RYBP\|BTBD7\|RBM19\|LSM14A\|ZNF3\|RYK | http://amigo.geneontology.org/amigo/term/GO:0007275 |
| positive regulation of protein ubiquitination | Gene Ontology | GO:0031398 | 5 | 72 | 0.002688 | 0.046489 | BIRC7\|BRCA1\|MAPK9\|PSMD10\|FANCI | http://amigo.geneontology.org/amigo/term/GO:0031398 |
| establishment or maintenance of epithelial cell apical/basal polarity | Gene Ontology | GO:0045197 | 3 | 20 | 0.002763 | 0.04654 | DLG1\|VANGL2\|CDX2 | http://amigo.geneontology.org/amigo/term/GO:0045197 |
| stress granule assembly | Gene Ontology | GO:0034063 | 3 | 20 | 0.002763 | 0.04654 | PUM2\|LSM14A\|G3BP1 | http://amigo.geneontology.org/amigo/term/GO:0034063 |
| intracellular estrogen receptor signaling pathway | Gene Ontology | GO:0030520 | 3 | 20 | 0.002763 | 0.04654 | DDX5\|DDX54\|DDX17 | http://amigo.geneontology.org/amigo/term/GO:0030520 |
| protein transport | Gene Ontology | GO:0015031 | 11 | 320 | 0.002993 | 0.048039 | RAB9A\|BBS9\|RAB35\|CORO7\|EXOC6B\|CCDC91\|FMN2\|SPIRE2\|CADPS\|SNX21\|EIF5A | http://amigo.geneontology.org/amigo/term/GO:0015031 |
| planar cell polarity pathway involved in axon guidance | Gene Ontology | GO:1904938 | 2 | 5 | 0.003071 | 0.048039 | VANGL2\|RYK | http://amigo.geneontology.org/amigo/term/GO:1904938 |
| membrane raft organization | Gene Ontology | GO:0031579 | 2 | 5 | 0.003071 | 0.048039 | DLG1\|POGZ | http://amigo.geneontology.org/amigo/term/GO:0031579 |
| thrombopoietin-mediated signaling | Gene Ontology | GO:0038163 | 2 | 5 | 0.003071 | 0.048039 | SPEN\|RBM15 | http://amigo.geneontology.org/amigo/term/GO:0038163 |

| | | | | | | | | |
|---|---|---|---|---|---|---|---|---|
| pathway | | | | | | | | |
| mRNA splice site selection | Gene Ontol-ogy | GO:0006376 | 3 | 21 | 0.003129 | 0.048039 | PTBP2\|RBMX\|LUC7L2 | http://amigo.geneontolo-gy.org/amigo/term/GO:0006376 |
| regulation of megakaryo-cyte differ-entiation | Gene Ontol-ogy | GO:0045652 | 4 | 45 | 0.003145 | 0.048039 | SPEN\|NFE2\|RBM15\|CNOT4 | http://amigo.geneontolo-gy.org/amigo/term/GO:0045652 |
| anatomical structure development | Gene Ontol-ogy | GO:0048856 | 4 | 45 | 0.003145 | 0.048039 | NR2C2\|NR2C1\|HECTD1\|SIX2 | http://amigo.geneontolo-gy.org/amigo/term/GO:0048856 |
| negative regulation of protein ki-nase B sig-naling | Gene Ontol-ogy | GO:0051898 | 4 | 46 | 0.003385 | 0.051319 | DLG1\|PTEN\|PHLPP1\|OTUD3 | http://amigo.geneontolo-gy.org/amigo/term/GO:0051898 |
| negative regulation of mRNA splic-ing, via spliceosome | Gene Ontol-ogy | GO:0048025 | 3 | 22 | 0.003523 | 0.051886 | DYRK1A\|HNRNPL\|RBMX | http://amigo.geneontolo-gy.org/amigo/term/GO:0048025 |
| regulation of cell size | Gene Ontol-ogy | GO:0008361 | 3 | 22 | 0.003523 | 0.051886 | VAV3\|VAV2\|MTOR | http://amigo.geneontolo-gy.org/amigo/term/GO:0008361 |
| aorta devel-opment | Gene Ontol-ogy | GO:0035904 | 3 | 22 | 0.003523 | 0.051886 | MYH10\|HECTD1\|DCTN5 | http://amigo.geneontolo-gy.org/amigo/term/GO:0035904 |
| positive regulation of cell adhesion | Gene Ontol-ogy | GO:0045785 | 4 | 48 | 0.003903 | 0.054231 | VAV3\|TNFSF18\|DUSP26\|BAG4 | http://amigo.geneontolo-gy.org/amigo/term/GO:0045785 |
| positive regulation of oligodendro-cyte differ-entiation | Gene Ontol-ogy | GO:0048714 | 3 | 23 | 0.003947 | 0.054231 | SHH\|MTOR\|TP73 | http://amigo.geneontolo-gy.org/amigo/term/GO:0048714 |
| negative regulation of cell migra-tion | Gene Ontol-ogy | GO:0030336 | 6 | 115 | 0.004019 | 0.054231 | PTEN\|TP53INP1\|NF1\|SHH\|MBD1\|RNF20 | http://amigo.geneontolo-gy.org/amigo/term/GO:0030336 |
| translational termination | Gene Ontol-ogy | GO:0006415 | 2 | 6 | 0.004062 | 0.054231 | APEH\|N6AMT1 | http://amigo.geneontolo-gy.org/amigo/term/GO:0006415 |
| regulation of fatty acid beta-oxidation | Gene Ontol-ogy | GO:0031998 | 2 | 6 | 0.004062 | 0.054231 | LONP2\|MTOR | http://amigo.geneontolo-gy.org/amigo/term/GO:0031998 |
| negative regulation of histone H3-K4 methyla-tion | Gene Ontol-ogy | GO:0051572 | 2 | 6 | 0.004062 | 0.054231 | BRCA1\|BCOR | http://amigo.geneontolo-gy.org/amigo/term/GO:0051572 |
| negative regulation of histone H3-K9 methyla-tion | Gene Ontol-ogy | GO:0051573 | 2 | 6 | 0.004062 | 0.054231 | BRCA1\|KDM3A | http://amigo.geneontolo-gy.org/amigo/term/GO:0051573 |
| muscular septum mor- | Gene Ontol- | GO:0003150 | 2 | 6 | 0.004062 | 0.054231 | VANGL2\|HEY2 | http://amigo.geneontolo- |

| | | | | | | | | |
|---|---|---|---|---|---|---|---|---|
| phogenesis | ogy | | | | | | | gy.org/amigo/term/GO:0003150 |
| vesicle tethering to Golgi | Gene Ontology | GO:0099041 | 2 | 6 | 0.004062 | 0.054231 | FAM91A1\|PHIP | http://amigo.geneontology.org/amigo/term/GO:0099041 |
| mRNA transcription | Gene Ontology | GO:0009299 | 2 | 6 | 0.004062 | 0.054231 | DDX5\|PPARD | http://amigo.geneontology.org/amigo/term/GO:0009299 |
| 'de novo' pyrimidine nucleobase biosynthetic process | Gene Ontology | GO:0006207 | 2 | 6 | 0.004062 | 0.054231 | MTOR\|CAD | http://amigo.geneontology.org/amigo/term/GO:0006207 |
| regulation of skeletal muscle cell differentiation | Gene Ontology | GO:2001014 | 2 | 6 | 0.004062 | 0.054231 | DDX5\|DDX17 | http://amigo.geneontology.org/amigo/term/GO:2001014 |
| positive regulation of striated muscle cell differentiation | Gene Ontology | GO:0051155 | 2 | 6 | 0.004062 | 0.054231 | SHH\|TRIM32 | http://amigo.geneontology.org/amigo/term/GO:0051155 |
| regulation of stem cell differentiation | Gene Ontology | GO:2000736 | 2 | 6 | 0.004062 | 0.054231 | KDM4C\|KDM3A | http://amigo.geneontology.org/amigo/term/GO:2000736 |
| regulation of apoptotic process | Gene Ontology | GO:0042981 | 8 | 197 | 0.004184 | 0.055293 | ANP32E\|BRCA1\|TP73\|RBM25\|TP53INP1\|PHLPP1\|USP36\|TARDBP | http://amigo.geneontology.org/amigo/term/GO:0042981 |
| positive regulation of cell growth | Gene Ontology | GO:0030307 | 5 | 81 | 0.00433 | 0.056926 | N6AMT1\|EIF4G2\|CDKN2AIP\|PSMD10\|TRIM32 | http://amigo.geneontology.org/amigo/term/GO:0030307 |
| regulation of cell morphogenesis | Gene Ontology | GO:0022604 | 3 | 24 | 0.0044 | 0.057266 | ZRANB1\|ZMYM4\|PHIP | http://amigo.geneontology.org/amigo/term/GO:0022604 |
| phospholipid biosynthetic process | Gene Ontology | GO:0008654 | 3 | 24 | 0.0044 | 0.057266 | MBOAT2\|PPARD\|TMEM38B | http://amigo.geneontology.org/amigo/term/GO:0008654 |
| negative regulation of autophagy | Gene Ontology | GO:0010507 | 4 | 50 | 0.004473 | 0.05792 | HERC1\|EIF4G2\|MTOR\|TLK2 | http://amigo.geneontology.org/amigo/term/GO:0010507 |
| cell cycle | Gene Ontology | GO:0007049 | 9 | 245 | 0.004613 | 0.059443 | CSNK1A1\|TLK2\|NEK1\|APPL1\|PCNP\|RNF8\|DBF4B\|DMTF1\|FANCI | http://amigo.geneontology.org/amigo/term/GO:0007049 |
| peptidyl-serine phosphorylation | Gene Ontology | GO:0018105 | 7 | 161 | 0.005075 | 0.064479 | CSNK1A1\|TLK2\|RPS6KB1\|MTOR\|DYRK1A\|CLK1\|MAPK9 | http://amigo.geneontology.org/amigo/term/GO:0018105 |
| nucleus localization | Gene Ontology | GO:0051647 | 2 | 7 | 0.005179 | 0.064479 | MTOR\|CLMN | http://amigo.geneontology.org/amigo/term/GO:0051647 |
| positive regulation of axon extension in- | Gene Ontology | GO:0048842 | 2 | 7 | 0.005179 | 0.064479 | CHD2\|BMPR2 | http://amigo.geneontology.org/amigo/term/GO:0048842 |

| | | | | | | | | |
|---|---|---|---|---|---|---|---|---|
| volved in axon guidance | | | | | | | | |
| positive regulation of RNA export from nucleus | Gene Ontology | GO:0046833 | 2 | 7 | 0.005179 | 0.064479 | RBM26\|CPSF6 | http://amigo.geneontology.org/amigo/term/GO:0046833 |
| vitamin A metabolic process | Gene Ontology | GO:0006776 | 2 | 7 | 0.005179 | 0.064479 | PPARD\|KDM5A | http://amigo.geneontology.org/amigo/term/GO:0006776 |
| mRNA cleavage | Gene Ontology | GO:0006379 | 2 | 7 | 0.005179 | 0.064479 | PCF11\|CSTF3 | http://amigo.geneontology.org/amigo/term/GO:0006379 |
| BMP signaling pathway | Gene Ontology | GO:0030509 | 5 | 85 | 0.00525 | 0.06473 | DDX5\|BMPR2\|ZFYVE16\|UBE2D3\|DLX5 | http://amigo.geneontology.org/amigo/term/GO:0030509 |
| positive regulation of cell population proliferation | Gene Ontology | GO:0008284 | 14 | 501 | 0.005333 | 0.064994 | DLG1\|ALDH3A1\|ROMO1\|PHIP\|CDX2\|FGF8\|KDM4C\|PTEN\|PURA\|CNOT8\|TIAL1\|DBF4B\|SHH\|REG3G | http://amigo.geneontology.org/amigo/term/GO:0008284 |
| regulation of DNA-binding transcription factor activity | Gene Ontology | GO:0051090 | 3 | 26 | 0.005397 | 0.064994 | CDKN2AIP\|MAPK9\|KDM5A | http://amigo.geneontology.org/amigo/term/GO:0051090 |
| protein catabolic process | Gene Ontology | GO:0030163 | 3 | 26 | 0.005397 | 0.064994 | MTOR\|CLN6\|AMBP | http://amigo.geneontology.org/amigo/term/GO:0030163 |
| protein phosphorylation | Gene Ontology | GO:0006468 | 13 | 451 | 0.00558 | 0.0666 | RPS6KA4\|RYK\|TLK2\|NEK1\|RPS6KB1\|CDKL2\|PRKD3\|BMPR2\|DYRK1A\|MTOR\|CLK2\|MAPK9\|CSNK1A1 | http://amigo.geneontology.org/amigo/term/GO:0006468 |
| protein K48-linked ubiquitination | Gene Ontology | GO:0070936 | 4 | 54 | 0.005773 | 0.067936 | TRIM32\|ARIH2\|RNF8\|UBE2D3 | http://amigo.geneontology.org/amigo/term/GO:0070936 |
| negative regulation of JNK cascade | Gene Ontology | GO:0046329 | 3 | 27 | 0.005943 | 0.069294 | MECOM\|ZNF675\|AMBP | http://amigo.geneontology.org/amigo/term/GO:0046329 |
| mRNA stabilization | Gene Ontology | GO:0048255 | 3 | 27 | 0.005943 | 0.069294 | MTOR\|HNRNPD\|TAF15 | http://amigo.geneontology.org/amigo/term/GO:0048255 |
| wound healing | Gene Ontology | GO:0042060 | 5 | 89 | 0.0063 | 0.070593 | VANGL2\|FGFR1OP2\|MTOR\|PPARD\|NF1 | http://amigo.geneontology.org/amigo/term/GO:0042060 |
| artery development | Gene Ontology | GO:0060840 | 2 | 8 | 0.006421 | 0.070593 | SHH\|BMPR2 | http://amigo.geneontology.org/amigo/term/GO:0060840 |
| nephron development | Gene Ontology | GO:0072006 | 2 | 8 | 0.006421 | 0.070593 | NUP160\|SIX2 | http://amigo.geneontology.org/amigo/term/GO:0072006 |
| heart valve development | Gene Ontology | GO:0003170 | 2 | 8 | 0.006421 | 0.070593 | HECTD1\|MDM4 | http://amigo.geneontology.org/amigo/te |

| | | | | | | | | |
|---|---|---|---|---|---|---|---|---|
| | | | | | | | | rm/GO:0003170 |
| negative regulation of mRNA polyadenylation | Gene Ontology | GO:1900364 | 2 | 8 | 0.006421 | 0.070593 | SUPT5H\|RNF20 | http://amigo.geneontology.org/amigo/term/GO:1900364 |
| protein processing | Gene Ontology | GO:0016485 | 4 | 56 | 0.006509 | 0.070593 | C2CD3\|OGT\|LONP2\|FKRP | http://amigo.geneontology.org/amigo/term/GO:0016485 |
| DNA damage response, signal transduction by p53 class mediator resulting in cell cycle arrest | Gene Ontology | GO:0006977 | 4 | 56 | 0.006509 | 0.070593 | CNOT8\|MDM4\|TFDP1\|CNOT4 | http://amigo.geneontology.org/amigo/term/GO:0006977 |
| phosphatidylcholine acyl-chain remodeling | Gene Ontology | GO:0036151 | 3 | 28 | 0.00652 | 0.070593 | MBOAT2\|NR1H2\|PLA2G4D | http://amigo.geneontology.org/amigo/term/GO:0036151 |
| positive regulation of mitotic nuclear division | Gene Ontology | GO:0045840 | 3 | 28 | 0.00652 | 0.070593 | IL1A\|FGF8\|PHIP | http://amigo.geneontology.org/amigo/term/GO:0045840 |
| negative regulation of fibroblast proliferation | Gene Ontology | GO:0048147 | 3 | 28 | 0.00652 | 0.070593 | TRIM32\|TP53INP1\|NF1 | http://amigo.geneontology.org/amigo/term/GO:0048147 |
| metanephros development | Gene Ontology | GO:0001656 | 3 | 29 | 0.007129 | 0.07623 | SHH\|NF1\|FGF8 | http://amigo.geneontology.org/amigo/term/GO:0001656 |
| protein K11-linked ubiquitination | Gene Ontology | GO:0070979 | 3 | 29 | 0.007129 | 0.07623 | ANAPC1\|RNF26\|UBE2D3 | http://amigo.geneontology.org/amigo/term/GO:0070979 |
| regulation of signal transduction by p53 class mediator | Gene Ontology | GO:1901796 | 6 | 131 | 0.007298 | 0.077716 | BRCA1\|TAF4\|TP73\|TP53INP1\|TAF15\|MDM4 | http://amigo.geneontology.org/amigo/term/GO:1901796 |
| autophagosome assembly | Gene Ontology | GO:0000045 | 4 | 59 | 0.007725 | 0.07893 | ATG9B\|ATG2B\|TP53INP1\|RAB35 | http://amigo.geneontology.org/amigo/term/GO:0000045 |
| mRNA 3'-end processing | Gene Ontology | GO:0031124 | 4 | 59 | 0.007725 | 0.07893 | PCF11\|PABPN1\|ZC3H11A\|CSTF3 | http://amigo.geneontology.org/amigo/term/GO:0031124 |
| cytolysis by host of symbiont cells | Gene Ontology | GO:0051838 | 2 | 9 | 0.007785 | 0.07893 | REG3G\|ROMO1 | http://amigo.geneontology.org/amigo/term/GO:0051838 |
| intestinal epithelial cell differentiation | Gene Ontology | GO:0060575 | 2 | 9 | 0.007785 | 0.07893 | CDX2\|CBFA2T2 | http://amigo.geneontology.org/amigo/term/GO:0060575 |
| histone H2B ubiquitination | Gene Ontology | GO:0033523 | 2 | 9 | 0.007785 | 0.07893 | RNF20\|RNF8 | http://amigo.geneontology.org/amigo/term/GO:0033523 |

| | | | | | | | | |
|---|---|---|---|---|---|---|---|---|
| bone marrow development | Gene Ontology | GO:0048539 | 2 | 9 | 0.007785 | 0.07893 | LRP5L\|ASXL1 | http://amigo.geneontology.org/amigo/term/GO:0048539 |
| negative regulation of cholesterol storage | Gene Ontology | GO:0010887 | 2 | 9 | 0.007785 | 0.07893 | PPARD\|NR1H2 | http://amigo.geneontology.org/amigo/term/GO:0010887 |
| regulation of chromosome segregation | Gene Ontology | GO:0051983 | 2 | 9 | 0.007785 | 0.07893 | PUM2\|ZNF207 | http://amigo.geneontology.org/amigo/term/GO:0051983 |
| sexual reproduction | Gene Ontology | GO:0019953 | 2 | 9 | 0.007785 | 0.07893 | VGF\|EIF4H | http://amigo.geneontology.org/amigo/term/GO:0019953 |
| interleukin-1-mediated signaling pathway | Gene Ontology | GO:0070498 | 5 | 94 | 0.00781 | 0.07893 | IL1A\|FBXW11\|RPS6KA4\|PSMD10\|IL1RAP | http://amigo.geneontology.org/amigo/term/GO:0070498 |
| negative regulation of epithelial cell proliferation | Gene Ontology | GO:0050680 | 4 | 60 | 0.008161 | 0.082153 | CDKN1C\|PTEN\|PPARD\|DLG1 | http://amigo.geneontology.org/amigo/term/GO:0050680 |
| germ cell development | Gene Ontology | GO:0007281 | 3 | 31 | 0.008446 | 0.083392 | FBXW11\|MTOR\|TIAL1 | http://amigo.geneontology.org/amigo/term/GO:0007281 |
| positive regulation of protein targeting to mitochondrion | Gene Ontology | GO:1903955 | 3 | 31 | 0.008446 | 0.083392 | ARIH2\|USP36\|UBE2D3 | http://amigo.geneontology.org/amigo/term/GO:1903955 |
| response to testosterone | Gene Ontology | GO:0033574 | 3 | 31 | 0.008446 | 0.083392 | TBXA2R\|HOXD13\|CAD | http://amigo.geneontology.org/amigo/term/GO:0033574 |
| positive regulation of DNA repair | Gene Ontology | GO:0045739 | 3 | 31 | 0.008446 | 0.083392 | BRCA1\|RNF8\|WRAP53 | http://amigo.geneontology.org/amigo/term/GO:0045739 |
| spinal cord development | Gene Ontology | GO:0021510 | 3 | 32 | 0.009154 | 0.084671 | PTBP2\|MTOR\|NF1 | http://amigo.geneontology.org/amigo/term/GO:0021510 |
| embryonic cranial skeleton morphogenesis | Gene Ontology | GO:0048701 | 3 | 32 | 0.009154 | 0.084671 | WDR19\|TBX15\|SIX2 | http://amigo.geneontology.org/amigo/term/GO:0048701 |
| proteasome-mediated ubiquitin-dependent protein catabolic process | Gene Ontology | GO:0043161 | 7 | 181 | 0.009185 | 0.084671 | FBXW11\|TBL1XR1\|SPOP\|UBE2D3\|PSMD10\|PCNP\|ARMC8 | http://amigo.geneontology.org/amigo/term/GO:0043161 |
| histone H3-K36 demethylation | Gene Ontology | GO:0070544 | 2 | 10 | 0.009266 | 0.084671 | KDM2A\|KDM4C | http://amigo.geneontology.org/amigo/term/GO:0070544 |
| cellular response to leucine | Gene Ontology | GO:0071233 | 2 | 10 | 0.009266 | 0.084671 | MTOR\|SESN3 | http://amigo.geneontology.org/amigo/term/GO:0071233 |

| | | | | | | | | |
|---|---|---|---|---|---|---|---|---|
| mitotic sister chromatid cohesion | Gene Ontology | GO:0007064 | 2 | 10 | 0.009266 | 0.084671 | POGZ\|PDS5A | http://amigo.geneontology.org/amigo/term/GO:0007064 |
| cellular response to leucine starvation | Gene Ontology | GO:1990253 | 2 | 10 | 0.009266 | 0.084671 | MTOR\|SESN3 | http://amigo.geneontology.org/amigo/term/GO:1990253 |
| histone exchange | Gene Ontology | GO:0043486 | 2 | 10 | 0.009266 | 0.084671 | ANP32E\|RNF8 | http://amigo.geneontology.org/amigo/term/GO:0043486 |
| ventricular cardiac muscle cell development | Gene Ontology | GO:0055015 | 2 | 10 | 0.009266 | 0.084671 | MYH10\|HEY2 | http://amigo.geneontology.org/amigo/term/GO:0055015 |
| regulation of bone resorption | Gene Ontology | GO:0045124 | 2 | 10 | 0.009266 | 0.084671 | IL20RA\|NF1 | http://amigo.geneontology.org/amigo/term/GO:0045124 |
| negative regulation of astrocyte differentiation | Gene Ontology | GO:0048712 | 2 | 10 | 0.009266 | 0.084671 | MBD1\|NF1 | http://amigo.geneontology.org/amigo/term/GO:0048712 |
| negative regulation of cell size | Gene Ontology | GO:0045792 | 2 | 10 | 0.009266 | 0.084671 | PTEN\|MTOR | http://amigo.geneontology.org/amigo/term/GO:0045792 |
| response to dietary excess | Gene Ontology | GO:0002021 | 2 | 10 | 0.009266 | 0.084671 | VGF\|TBL1XR1 | http://amigo.geneontology.org/amigo/term/GO:0002021 |
| regulation of small GTPase mediated signal transduction | Gene Ontology | GO:0051056 | 6 | 139 | 0.009515 | 0.08619 | SOS2\|ARHGEF18\|VAV3\|VAV2\|FAM13A\|ARHGAP18 | http://amigo.geneontology.org/amigo/term/GO:0051056 |
| synapse assembly | Gene Ontology | GO:0007416 | 4 | 63 | 0.009566 | 0.08619 | PTEN\|SDK2\|CHD2\|RYK | http://amigo.geneontology.org/amigo/term/GO:0007416 |
| regulation of translation | Gene Ontology | GO:0006417 | 4 | 63 | 0.009566 | 0.08619 | PUM2\|PTBP2\|LSM14A\|HNRNPL | http://amigo.geneontology.org/amigo/term/GO:0006417 |
| heart looping | Gene Ontology | GO:0001947 | 4 | 63 | 0.009566 | 0.08619 | SHH\|C2CD3\|VANGL2\|FGF8 | http://amigo.geneontology.org/amigo/term/GO:0001947 |
| actin filament polymerization | Gene Ontology | GO:0030041 | 3 | 33 | 0.009896 | 0.087639 | TTC17\|CORO7\|SPIRE2 | http://amigo.geneontology.org/amigo/term/GO:0030041 |
| positive regulation of macroautophagy | Gene Ontology | GO:0016239 | 3 | 33 | 0.009896 | 0.087639 | SUPT5H\|PAFAH1B2\|SESN3 | http://amigo.geneontology.org/amigo/term/GO:0016239 |
| lamellipodium assembly | Gene Ontology | GO:0030032 | 3 | 33 | 0.009896 | 0.087639 | SPATA13\|VAV3\|VAV2 | http://amigo.geneontology.org/amigo/term/GO:0030032 |
| ventricular septum de- | Gene Ontol- | GO:0003281 | 3 | 33 | 0.009896 | 0.087639 | HECTD1\|MDM4\|DCTN5 | http://amigo.geneontolo- |

| | | | | | | | | |
|---|---|---|---|---|---|---|---|---|
| velopment | ogy | | | | | | | gy.org/amigo/te rm/GO:0003281 |
| rRNA pro-cessing | Gene Ontol-ogy | GO:00 06364 | 6 | 141 | 0.010136 | 0.089149 | DDX17\|HELQ\|RRP1B\|RCL1\|D DX54\|NOL9 | http://amigo.gen eontolo-gy.org/amigo/te rm/GO:0006364 |
| brain devel-opment | Gene Ontol-ogy | GO:00 07420 | 8 | 231 | 0.010184 | 0.089268 | BPTF\|C2CD3\|RAB3GAP1\|KC NAB1\|BMPR2\|NF1\|MTOR\|PA FAH1B2 | http://amigo.gen eontolo-gy.org/amigo/te rm/GO:0007420 |
| peptidyl-threonine phosphoryla-tion | Gene Ontol-ogy | GO:00 18107 | 4 | 65 | 0.010584 | 0.090875 | DYRK1A\|CLK1\|MTOR\|CAD | http://amigo.gen eontolo-gy.org/amigo/te rm/GO:0018107 |
| anoikis | Gene Ontol-ogy | GO:00 43276 | 2 | 11 | 0.010861 | 0.090875 | MTOR\|TFDP1 | http://amigo.gen eontolo-gy.org/amigo/te rm/GO:0043276 |
| response to amine | Gene Ontol-ogy | GO:00 14075 | 2 | 11 | 0.010861 | 0.090875 | RRM2B\|CAD | http://amigo.gen eontolo-gy.org/amigo/te rm/GO:0014075 |
| retrograde axonal transport | Gene Ontol-ogy | GO:00 08090 | 2 | 11 | 0.010861 | 0.090875 | TMEM108\|FBXW11 | http://amigo.gen eontolo-gy.org/amigo/te rm/GO:0008090 |
| postreplica-tion repair | Gene Ontol-ogy | GO:00 06301 | 2 | 11 | 0.010861 | 0.090875 | BRCA1\|NSMCE1 | http://amigo.gen eontolo-gy.org/amigo/te rm/GO:0006301 |
| positive regulation of embryonic development | Gene Ontol-ogy | GO:00 40019 | 2 | 11 | 0.010861 | 0.090875 | NR2C2\|RBM19 | http://amigo.gen eontolo-gy.org/amigo/te rm/GO:0040019 |
| rhodopsin mediated signaling pathway | Gene Ontol-ogy | GO:00 16056 | 2 | 11 | 0.010861 | 0.090875 | CNGB1\|PDE6A | http://amigo.gen eontolo-gy.org/amigo/te rm/GO:0016056 |
| gonad devel-opment | Gene Ontol-ogy | GO:00 08406 | 2 | 11 | 0.010861 | 0.090875 | WDR19\|FGF8 | http://amigo.gen eontolo-gy.org/amigo/te rm/GO:0008406 |
| branching involved in salivary gland mor-phogenesis | Gene Ontol-ogy | GO:00 60445 | 2 | 11 | 0.010861 | 0.090875 | SHH\|FGF8 | http://amigo.gen eontolo-gy.org/amigo/te rm/GO:0060445 |
| cellular re-sponse to nerve growth factor stimu-lus | Gene Ontol-ogy | GO:19 90090 | 3 | 35 | 0.011483 | 0.095763 | PTEN\|RAB35\|CDC5L | http://amigo.gen eontolo-gy.org/amigo/te rm/GO:1990090 |
| positive regulation of gene expres-sion | Gene Ontol-ogy | GO:00 10628 | 11 | 391 | 0.01214 | 0.100917 | BRCA1\|RAB3GAP1\|MED23\|F UBP1\|KDM4C\|PTEN\|MTOR\|F GF8\|IL1A\|MAPK9\|HEY2 | http://amigo.gen eontolo-gy.org/amigo/te rm/GO:0010628 |
| mRNA poly-adenylation | Gene Ontol-ogy | GO:00 06378 | 3 | 36 | 0.012327 | 0.101217 | PCF11\|PAPOLG\|CSTF3 | http://amigo.gen eontolo-gy.org/amigo/te rm/GO:0006378 |
| placenta blood vessel development | Gene Ontol-ogy | GO:00 60674 | 2 | 12 | 0.012569 | 0.101217 | SPEN\|RBM15 | http://amigo.gen eontolo-gy.org/amigo/te rm/GO:0060674 |

| | | | | | | | | |
|---|---|---|---|---|---|---|---|---|
| regulation of telomere maintenance | Gene Ontol-ogy | GO:0032204 | 2 | 12 | 0.012569 | 0.101217 | YLPM1\|HNRNPD | http://amigo.geneontology.org/amigo/term/GO:0032204 |
| actin filament network formation | Gene Ontol-ogy | GO:0051639 | 2 | 12 | 0.012569 | 0.101217 | PLS3\|SPIRE2 | http://amigo.geneontology.org/amigo/term/GO:0051639 |
| head development | Gene Ontol-ogy | GO:0060322 | 2 | 12 | 0.012569 | 0.101217 | EDNRA\|PHIP | http://amigo.geneontology.org/amigo/term/GO:0060322 |
| histone H2A monoubiquitination | Gene Ontol-ogy | GO:0035518 | 2 | 12 | 0.012569 | 0.101217 | RYBP\|BCOR | http://amigo.geneontology.org/amigo/term/GO:0035518 |
| positive regulation of smoothened signaling pathway | Gene Ontol-ogy | GO:0045880 | 3 | 37 | 0.013207 | 0.106026 | SHH\|SCUBE3\|TXNDC15 | http://amigo.geneontology.org/amigo/term/GO:0045880 |
| negative regulation of Notch signaling pathway | Gene Ontol-ogy | GO:0045746 | 3 | 38 | 0.014121 | 0.11061 | METTL3\|HEY2\|CBFA2T2 | http://amigo.geneontology.org/amigo/term/GO:0045746 |
| regulation of epithelial to mesenchymal transition | Gene Ontol-ogy | GO:0010717 | 2 | 13 | 0.014385 | 0.11061 | PHLDB2\|POFUT2 | http://amigo.geneontology.org/amigo/term/GO:0010717 |
| epithelial to mesenchymal transition involved in endocardial cushion formation | Gene Ontol-ogy | GO:0003198 | 2 | 13 | 0.014385 | 0.11061 | HEY2\|FGF8 | http://amigo.geneontology.org/amigo/term/GO:0003198 |
| regulation of DNA methylation | Gene Ontol-ogy | GO:0044030 | 2 | 13 | 0.014385 | 0.11061 | BRCA1\|MBD1 | http://amigo.geneontology.org/amigo/term/GO:0044030 |
| regulation of insulin receptor signaling pathway | Gene Ontol-ogy | GO:0046626 | 2 | 13 | 0.014385 | 0.11061 | OGT\|SESN3 | http://amigo.geneontology.org/amigo/term/GO:0046626 |
| regulation of type I interferon production | Gene Ontol-ogy | GO:0032479 | 2 | 13 | 0.014385 | 0.11061 | TRIM32\|RNF26 | http://amigo.geneontology.org/amigo/term/GO:0032479 |
| atrioventricular valve morphogenesis | Gene Ontol-ogy | GO:0003181 | 2 | 13 | 0.014385 | 0.11061 | MDM4\|BMPR2 | http://amigo.geneontology.org/amigo/term/GO:0003181 |
| oligosaccharide biosynthetic process | Gene Ontol-ogy | GO:0009312 | 2 | 13 | 0.014385 | 0.11061 | MPDU1\|B3GALNT1 | http://amigo.geneontology.org/amigo/term/GO:0009312 |
| atrial septum morphogenesis | Gene Ontol-ogy | GO:0060413 | 2 | 13 | 0.014385 | 0.11061 | BMPR2\|HEY2 | http://amigo.geneontology.org/amigo/term/GO:0060413 |
| histone deacetylation | Gene Ontol- | GO:0016575 | 3 | 39 | 0.015071 | 0.11356 | RCOR3\|TBL1XR1\|SUDS3 | http://amigo.geneontolo- |

| | | | | | | | | |
|---|---|---|---|---|---|---|---|---|
| | ogy | | | | | | | gy.org/amigo/term/GO:0016575 |
| embryonic limb morphogenesis | Gene Ontology | GO:0030326 | 3 | 39 | 0.015071 | 0.11356 | SHH\|DLX5\|WDR19 | http://amigo.geneontology.org/amigo/term/GO:0030326 |
| negative regulation of signaling receptor activity | Gene Ontology | GO:2000272 | 3 | 39 | 0.015071 | 0.11356 | PTEN\|DKK4\|LY6H | http://amigo.geneontology.org/amigo/term/GO:2000272 |
| circadian rhythm | Gene Ontology | GO:0007623 | 4 | 74 | 0.016023 | 0.118707 | DYRK1A\|HNRNPR\|METTL3\|HNRNPL | http://amigo.geneontology.org/amigo/term/GO:0007623 |
| glycosphingolipid metabolic process | Gene Ontology | GO:0006687 | 3 | 40 | 0.016056 | 0.118707 | NEU2\|ESYT2\|B3GALNT1 | http://amigo.geneontology.org/amigo/term/GO:0006687 |
| termination of RNA polymerase II transcription | Gene Ontology | GO:0006369 | 3 | 40 | 0.016056 | 0.118707 | PCF11\|PABPN1\|CSTF3 | http://amigo.geneontology.org/amigo/term/GO:0006369 |
| cellular response to ethanol | Gene Ontology | GO:0071361 | 2 | 14 | 0.016307 | 0.118707 | PTEN\|TP53INP1 | http://amigo.geneontology.org/amigo/term/GO:0071361 |
| Rab protein signal transduction | Gene Ontology | GO:0032482 | 2 | 14 | 0.016307 | 0.118707 | RAB9A\|RAB35 | http://amigo.geneontology.org/amigo/term/GO:0032482 |
| white fat cell differentiation | Gene Ontology | GO:0050872 | 2 | 14 | 0.016307 | 0.118707 | CTBP2\|TBL1XR1 | http://amigo.geneontology.org/amigo/term/GO:0050872 |
| production of miRNAs involved in gene silencing by miRNA | Gene Ontology | GO:0035196 | 2 | 14 | 0.016307 | 0.118707 | PUM2\|ZC3H7B | http://amigo.geneontology.org/amigo/term/GO:0035196 |
| corpus callosum development | Gene Ontology | GO:0022038 | 2 | 14 | 0.016307 | 0.118707 | HERC1\|RYK | http://amigo.geneontology.org/amigo/term/GO:0022038 |
| sex differentiation | Gene Ontology | GO:0007548 | 2 | 14 | 0.016307 | 0.118707 | TSPY1\|CDKL2 | http://amigo.geneontology.org/amigo/term/GO:0007548 |
| cellular response to hypoxia | Gene Ontology | GO:0071456 | 5 | 114 | 0.016355 | 0.118727 | MTOR\|PTEN\|PPARD\|MDM4\|FMN2 | http://amigo.geneontology.org/amigo/term/GO:0071456 |
| response to nutrient levels | Gene Ontology | GO:0031667 | 3 | 41 | 0.017076 | 0.122916 | MTOR\|RPS6KB1\|MBD1 | http://amigo.geneontology.org/amigo/term/GO:0031667 |
| Golgi to endosome transport | Gene Ontology | GO:0006895 | 2 | 15 | 0.018331 | 0.12837 | AP2A1\|CORO7 | http://amigo.geneontology.org/amigo/term/GO:0006895 |
| hematopoietic stem cell proliferation | Gene Ontology | GO:0071425 | 2 | 15 | 0.018331 | 0.12837 | MECOM\|ARIH2 | http://amigo.geneontology.org/amigo/te |

| | | | | | | | | |
|---|---|---|---|---|---|---|---|---|
| | | | | | | | | rm/GO:0071425 |
| adenylate cyclase-activating adrenergic receptor signaling pathway | Gene Ontol-ogy | GO:0071880 | 2 | 15 | 0.018331 | 0.12837 | ADRB3\|ADRA1B | http://amigo.geneontolo-gy.org/amigo/term/GO:0071880 |
| dolichol-linked oligo-saccharide biosynthetic process | Gene Ontol-ogy | GO:0006488 | 2 | 15 | 0.018331 | 0.12837 | ALG10B\|MPDU1 | http://amigo.geneontolo-gy.org/amigo/term/GO:0006488 |
| negative regulation of cardiac mus-cle cell pro-liferation | Gene Ontol-ogy | GO:0060044 | 2 | 15 | 0.018331 | 0.12837 | PTEN\|TP73 | http://amigo.geneontolo-gy.org/amigo/term/GO:0060044 |
| negative regulation of DNA dam-age re-sponse, sig-nal transduc-tion by p53 class media-tor | Gene Ontol-ogy | GO:0043518 | 2 | 15 | 0.018331 | 0.12837 | DYRK1A\|PSMD10 | http://amigo.geneontolo-gy.org/amigo/term/GO:0043518 |
| energy re-serve meta-bolic process | Gene Ontol-ogy | GO:0006112 | 2 | 15 | 0.018331 | 0.12837 | ADRB3\|MTOR | http://amigo.geneontolo-gy.org/amigo/term/GO:0006112 |
| regulation of synaptic transmission, GABAergic | Gene Ontol-ogy | GO:0032228 | 2 | 15 | 0.018331 | 0.12837 | PTEN\|NF1 | http://amigo.geneontolo-gy.org/amigo/term/GO:0032228 |
| receptor-mediated endocytosis | Gene Ontol-ogy | GO:0006898 | 6 | 163 | 0.01898 | 0.132554 | TMEM108\|IGKV1-5\|FPR2\|AMBP\|ADRB3\|AP2A1 | http://amigo.geneontolo-gy.org/amigo/term/GO:0006898 |
| negative regulation of nucleic acid-templated transcription | Gene Ontol-ogy | GO:1903507 | 3 | 43 | 0.019222 | 0.133519 | RCOR3\|CDYL2\|DDX54 | http://amigo.geneontolo-gy.org/amigo/term/GO:1903507 |
| protein im-port into nucleus | Gene Ontol-ogy | GO:0006606 | 4 | 79 | 0.019681 | 0.134909 | APPL1\|IPO9\|SIX2\|PPP1R10 | http://amigo.geneontolo-gy.org/amigo/term/GO:0006606 |
| negative regulation of translation | Gene Ontol-ogy | GO:0017148 | 4 | 79 | 0.019681 | 0.134909 | TIA1\|PURA\|CNOT8\|CAPRIN1 | http://amigo.geneontolo-gy.org/amigo/term/GO:0017148 |
| biologi-cal_process | Gene Ontol-ogy | GO:0008150 | 14 | 594 | 0.020246 | 0.134909 | ZMYM2\|LAGE3\|NYX\|TMEM131\|RCL1\|C6orf163\|RUFY2\|ZNF862\|KRTDAP\|RUSC2\|YLPM1\|TMEM108\|WSB1\|ZNF785 | http://amigo.geneontolo-gy.org/amigo/term/GO:0008150 |
| protein K63-linked ubiq-uitination | Gene Ontol-ogy | GO:0070534 | 3 | 44 | 0.020349 | 0.134909 | HECTD1\|ARIH2\|RNF8 | http://amigo.geneontolo-gy.org/amigo/term/GO:0070534 |
| cellular re-sponse to amino acid starvation | Gene Ontol-ogy | GO:0034198 | 3 | 44 | 0.020349 | 0.134909 | MTOR\|HNRNPL\|SESN3 | http://amigo.geneontolo-gy.org/amigo/term/GO:0034198 |

| | | | | | | | | |
|---|---|---|---|---|---|---|---|---|
| thymus development | Gene Ontology | GO:0048538 | 3 | 44 | 0.020349 | 0.134909 | SHH\|ASXL1\|ZBTB1 | http://amigo.geneontology.org/amigo/term/GO:0048538 |
| intracellular signal transduction | Gene Ontology | GO:0035556 | 10 | 369 | 0.020448 | 0.134909 | ASB10\|ADRA1B\|RPS6KA4\|TLK2\|RPS6KB1\|FMN2\|WSB1\|MAPK9\|RASA1\|NSMCE1 | http://amigo.geneontology.org/amigo/term/GO:0035556 |
| cellular response to nitric oxide | Gene Ontology | GO:0071732 | 2 | 16 | 0.020456 | 0.134909 | HNRNPD\|MBD1 | http://amigo.geneontology.org/amigo/term/GO:0071732 |
| regulation of mRNA splicing, via spliceosome | Gene Ontology | GO:0048024 | 2 | 16 | 0.020456 | 0.134909 | RBM39\|TIA1 | http://amigo.geneontology.org/amigo/term/GO:0048024 |
| mRNA destabilization | Gene Ontology | GO:0061157 | 2 | 16 | 0.020456 | 0.134909 | HNRNPR\|METTL3 | http://amigo.geneontology.org/amigo/term/GO:0061157 |
| endothelial cell proliferation | Gene Ontology | GO:0001935 | 2 | 16 | 0.020456 | 0.134909 | DLG1\|BMPR2 | http://amigo.geneontology.org/amigo/term/GO:0001935 |
| dentate gyrus development | Gene Ontology | GO:0021542 | 2 | 16 | 0.020456 | 0.134909 | PTEN\|TMEM108 | http://amigo.geneontology.org/amigo/term/GO:0021542 |
| epidermis development | Gene Ontology | GO:0008544 | 4 | 80 | 0.020469 | 0.134909 | TFDP1\|KRTDAP\|SATB1\|KDM5A | http://amigo.geneontology.org/amigo/term/GO:0008544 |
| cellular response to calcium ion | Gene Ontology | GO:0071277 | 4 | 81 | 0.021277 | 0.139873 | CDKN2AIP\|MBD1\|MEF2A\|CPNE5 | http://amigo.geneontology.org/amigo/term/GO:0071277 |
| protein N-linked glycosylation | Gene Ontology | GO:0006487 | 3 | 45 | 0.021511 | 0.140457 | TMEM165\|ALG10B\|MGAT4A | http://amigo.geneontology.org/amigo/term/GO:0006487 |
| response to ionizing radiation | Gene Ontology | GO:0010212 | 3 | 45 | 0.021511 | 0.140457 | BRCA1\|CLK2\|RNF8 | http://amigo.geneontology.org/amigo/term/GO:0010212 |
| peptidyl-tyrosine phosphorylation | Gene Ontology | GO:0018108 | 5 | 124 | 0.022332 | 0.143593 | DYRK1A\|CLK1\|CLK2\|RYK\|NEK1 | http://amigo.geneontology.org/amigo/term/GO:0018108 |
| phosphate-containing compound metabolic process | Gene Ontology | GO:0006796 | 2 | 17 | 0.022678 | 0.143593 | SLC17A4\|INPP1 | http://amigo.geneontology.org/amigo/term/GO:0006796 |
| long-term synaptic depression | Gene Ontology | GO:0060292 | 2 | 17 | 0.022678 | 0.143593 | PTEN\|SLC24A1 | http://amigo.geneontology.org/amigo/term/GO:0060292 |
| inner ear receptor cell stereocilium organization | Gene Ontology | GO:0060122 | 2 | 17 | 0.022678 | 0.143593 | VANGL2\|FAT4 | http://amigo.geneontology.org/amigo/term/GO:0060122 |
| 3'-UTR-mediated mRNA destabilization | Gene Ontology | GO:0061158 | 2 | 17 | 0.022678 | 0.143593 | HNRNPD\|TARDBP | http://amigo.geneontology.org/amigo/term/GO:0061158 |

| | | | | | | | | |
|---|---|---|---|---|---|---|---|---|
| cardiac muscle tissue development | Gene Ontology | GO:0048738 | 2 | 17 | 0.022678 | 0.143593 | PTEN\|BMPR2 | http://amigo.geneontology.org/amigo/term/GO:0048738 |
| positive regulation of cartilage development | Gene Ontology | GO:0061036 | 2 | 17 | 0.022678 | 0.143593 | BMPR2\|TAPT1 | http://amigo.geneontology.org/amigo/term/GO:0061036 |
| regulation of protein stability | Gene Ontology | GO:0031647 | 4 | 83 | 0.02295 | 0.14487 | PTEN\|CDKN2AIP\|USP36\|TARDBP | http://amigo.geneontology.org/amigo/term/GO:0031647 |
| negative regulation of cell growth | Gene Ontology | GO:0030308 | 5 | 125 | 0.022998 | 0.14487 | TCHP\|CDKN2AIP\|ST20\|BMPR2\|PPARD | http://amigo.geneontology.org/amigo/term/GO:0030308 |
| Fc-epsilon receptor signaling pathway | Gene Ontology | GO:0038095 | 6 | 171 | 0.023202 | 0.145479 | FBXW11\|IGKV1-5\|VAV3\|VAV2\|PSMD10\|MAPK9 | http://amigo.geneontology.org/amigo/term/GO:0038095 |
| cholesterol homeostasis | Gene Ontology | GO:0042632 | 4 | 84 | 0.023815 | 0.147958 | LRP5L\|POGZ\|MED13\|NR1H2 | http://amigo.geneontology.org/amigo/term/GO:0042632 |
| inositol phosphate metabolic process | Gene Ontology | GO:0043647 | 3 | 47 | 0.023942 | 0.147958 | PTEN\|INPP1\|INPP5A | http://amigo.geneontology.org/amigo/term/GO:0043647 |
| positive regulation of cell division | Gene Ontology | GO:0051781 | 3 | 47 | 0.023942 | 0.147958 | SHH\|IL1A\|FGF8 | http://amigo.geneontology.org/amigo/term/GO:0051781 |
| bone development | Gene Ontology | GO:0060348 | 3 | 47 | 0.023942 | 0.147958 | PLS3\|TMEM38B\|FGF8 | http://amigo.geneontology.org/amigo/term/GO:0060348 |
| response to activity | Gene Ontology | GO:0014823 | 3 | 47 | 0.023942 | 0.147958 | PTEN\|PPARD\|MTOR | http://amigo.geneontology.org/amigo/term/GO:0014823 |
| inflammatory response | Gene Ontology | GO:0006954 | 10 | 381 | 0.024676 | 0.150496 | TBXA2R\|RPS6KA4\|ORM1\|IL18RAP\|NFX1\|FPR2\|LTB4R\|MBD1\|IL1RAP\|IL1A | http://amigo.geneontology.org/amigo/term/GO:0006954 |
| actin filament-based movement | Gene Ontology | GO:0030048 | 2 | 18 | 0.024995 | 0.150496 | MYH10\|MYO1B | http://amigo.geneontology.org/amigo/term/GO:0030048 |
| negative regulation of myeloid cell differentiation | Gene Ontology | GO:0045638 | 2 | 18 | 0.024995 | 0.150496 | SPEN\|RBM15 | http://amigo.geneontology.org/amigo/term/GO:0045638 |
| negative regulation of focal adhesion assembly | Gene Ontology | GO:0051895 | 2 | 18 | 0.024995 | 0.150496 | PTEN\|PHLDB2 | http://amigo.geneontology.org/amigo/term/GO:0051895 |
| alternative mRNA splicing, via spliceosome | Gene Ontology | GO:0000380 | 2 | 18 | 0.024995 | 0.150496 | DDX5\|DDX17 | http://amigo.geneontology.org/amigo/term/GO:0000380 |
| glutamine metabolic process | Gene Ontology | GO:0006541 | 2 | 18 | 0.024995 | 0.150496 | GMPS\|CAD | http://amigo.geneontology.org/amigo/te |

| | | | | | | | | |
|---|---|---|---|---|---|---|---|---|
| | | | | | | | | rm/GO:0006541 |
| embryonic digestive tract morphogenesis | Gene Ontology | GO:0048557 | 2 | 18 | 0.024995 | 0.150496 | SHH\|SIX2 | http://amigo.geneontology.org/amigo/term/GO:0048557 |
| positive regulation of histone acetylation | Gene Ontology | GO:0035066 | 2 | 18 | 0.024995 | 0.150496 | BRCA1\|RPS6KA4 | http://amigo.geneontology.org/amigo/term/GO:0035066 |
| phosphatidylglycerol acyl-chain remodeling | Gene Ontology | GO:0036148 | 2 | 18 | 0.024995 | 0.150496 | MBOAT2\|PLA2G4D | http://amigo.geneontology.org/amigo/term/GO:0036148 |
| generation of precursor metabolites and energy | Gene Ontology | GO:0006091 | 3 | 48 | 0.025211 | 0.151443 | VGF\|ADRB3\|PPARD | http://amigo.geneontology.org/amigo/term/GO:0006091 |
| ephrin receptor signaling pathway | Gene Ontology | GO:0048013 | 4 | 86 | 0.025605 | 0.15345 | VAV3\|VAV2\|AP2A1\|RASA1 | http://amigo.geneontology.org/amigo/term/GO:0048013 |
| protein-containing complex assembly | Gene Ontology | GO:0065003 | 5 | 129 | 0.025789 | 0.153839 | MDM4\|WDR19\|TUBGCP4\|MAX\|IL1RAP | http://amigo.geneontology.org/amigo/term/GO:0065003 |
| negative regulation of canonical Wnt signaling pathway | Gene Ontology | GO:0090090 | 6 | 176 | 0.026138 | 0.155559 | ANKRD6\|CSNK1A1\|DKK4\|PSMD10\|G3BP1\|SHH | http://amigo.geneontology.org/amigo/term/GO:0090090 |
| cell cycle arrest | Gene Ontology | GO:0007050 | 5 | 130 | 0.026519 | 0.156758 | CDKN1C\|EIF4G2\|TP53INP1\|TP73\|MTOR | http://amigo.geneontology.org/amigo/term/GO:0007050 |
| protein import into mitochondrial matrix | Gene Ontology | GO:0030150 | 2 | 19 | 0.027404 | 0.157985 | ROMO1\|TIMM23 | http://amigo.geneontology.org/amigo/term/GO:0030150 |
| osteoblast development | Gene Ontology | GO:0002076 | 2 | 19 | 0.027404 | 0.157985 | SHH\|LRP5L | http://amigo.geneontology.org/amigo/term/GO:0002076 |
| positive regulation of monocyte chemotaxis | Gene Ontology | GO:0090026 | 2 | 19 | 0.027404 | 0.157985 | MOSPD2\|FPR2 | http://amigo.geneontology.org/amigo/term/GO:0090026 |
| TOR signaling | Gene Ontology | GO:0031929 | 2 | 19 | 0.027404 | 0.157985 | MTOR\|RPS6KB1 | http://amigo.geneontology.org/amigo/term/GO:0031929 |
| negative regulation of cell-matrix adhesion | Gene Ontology | GO:0001953 | 2 | 19 | 0.027404 | 0.157985 | RASA1\|NF1 | http://amigo.geneontology.org/amigo/term/GO:0001953 |
| post-anal tail morphogenesis | Gene Ontology | GO:0036342 | 2 | 19 | 0.027404 | 0.157985 | VANGL2\|SFTPC | http://amigo.geneontology.org/amigo/term/GO:0036342 |
| protein localization to centrosome | Gene Ontology | GO:0071539 | 2 | 19 | 0.027404 | 0.157985 | DCTN2\|C2CD3 | http://amigo.geneontology.org/amigo/term/GO:0071539 |
| telomere maintenance | Gene Ontol- | GO:0007004 | 2 | 19 | 0.027404 | 0.157985 | CNGB1\|WRAP53 | http://amigo.geneontolo- |

| | | | | | | | | |
|---|---|---|---|---|---|---|---|---|
| via telomer-ase | ogy | | | | | | | gy.org/amigo/term/GO:0007004 |
| response to drug | Gene Ontology | GO:0042493 | 8 | 280 | 0.027702 | 0.159346 | TBXA2R\|VAV3\|ALDH3A1\|TP73\|POGZ\|PTEN\|MBD1\|FGF8 | http://amigo.geneontology.org/amigo/term/GO:0042493 |
| interstrand cross-link repair | Gene Ontology | GO:0036297 | 3 | 50 | 0.027856 | 0.159875 | MUS81\|RNF8\|FANCI | http://amigo.geneontology.org/amigo/term/GO:0036297 |
| positive regulation of apoptotic process | Gene Ontology | GO:0043065 | 9 | 335 | 0.028327 | 0.16222 | SOS2\|RRP1B\|VAV3\|VAV2\|SUDS3\|ARHGEF18\|TP53INP1\|NF1\|RYBP | http://amigo.geneontology.org/amigo/term/GO:0043065 |
| positive regulation of actin filament polymerization | Gene Ontology | GO:0030838 | 3 | 51 | 0.029231 | 0.165357 | DLG1\|MTOR\|BAG4 | http://amigo.geneontology.org/amigo/term/GO:0030838 |
| stem cell population maintenance | Gene Ontology | GO:0019827 | 3 | 51 | 0.029231 | 0.165357 | KDM4C\|MED28\|METTL3 | http://amigo.geneontology.org/amigo/term/GO:0019827 |
| cellular response to UV | Gene Ontology | GO:0034644 | 3 | 51 | 0.029231 | 0.165357 | ZBTB1\|METTL3\|TP53INP1 | http://amigo.geneontology.org/amigo/term/GO:0034644 |
| cellular response to amino acid stimulus | Gene Ontology | GO:0071230 | 3 | 51 | 0.029231 | 0.165357 | MTOR\|HNRNPD\|SESN3 | http://amigo.geneontology.org/amigo/term/GO:0071230 |
| animal organ morphogenesis | Gene Ontology | GO:0009887 | 5 | 134 | 0.02957 | 0.165357 | ASXL1\|FGF8\|CDX2\|ETV7\|LY6H | http://amigo.geneontology.org/amigo/term/GO:0009887 |
| negative regulation of MAPK cascade | Gene Ontology | GO:0043409 | 2 | 20 | 0.029902 | 0.165357 | PSMD10\|NF1 | http://amigo.geneontology.org/amigo/term/GO:0043409 |
| regulation of myelination | Gene Ontology | GO:0031641 | 2 | 20 | 0.029902 | 0.165357 | DLG1\|MTOR | http://amigo.geneontology.org/amigo/term/GO:0031641 |
| poly(A)+ mRNA export from nucleus | Gene Ontology | GO:0016973 | 2 | 20 | 0.029902 | 0.165357 | PABPN1\|ZC3H11A | http://amigo.geneontology.org/amigo/term/GO:0016973 |
| protein tetramerization | Gene Ontology | GO:0051262 | 2 | 20 | 0.029902 | 0.165357 | CPSF6\|TP73 | http://amigo.geneontology.org/amigo/term/GO:0051262 |
| positive regulation of CREB transcription factor activity | Gene Ontology | GO:0032793 | 2 | 20 | 0.029902 | 0.165357 | MAML2\|RPS6KA4 | http://amigo.geneontology.org/amigo/term/GO:0032793 |
| positive regulation of transcription initiation from RNA polymerase II promoter | Gene Ontology | GO:0060261 | 2 | 20 | 0.029902 | 0.165357 | MED13\|MED17 | http://amigo.geneontology.org/amigo/term/GO:0060261 |

| | | | | | | | | |
|---|---|---|---|---|---|---|---|---|
| peroxisome organization | Gene Ontol-ogy | GO:0007031 | 2 | 20 | 0.029902 | 0.165357 | PEX11A\|LONP2 | http://amigo.geneontology.org/amigo/term/GO:0007031 |
| endocytosis | Gene Ontol-ogy | GO:0006897 | 6 | 182 | 0.029974 | 0.165401 | ESYT2\|POGZ\|AP2A1\|ATP9B\|CLINT1\|SHH | http://amigo.geneontology.org/amigo/term/GO:0006897 |
| negative regulation of catalytic activity | Gene Ontol-ogy | GO:0043086 | 3 | 52 | 0.030642 | 0.16768 | HNRNPR\|ANP32E\|PPP1R12A | http://amigo.geneontology.org/amigo/term/GO:0043086 |
| mitotic cyto-kinesis | Gene Ontol-ogy | GO:0000281 | 3 | 52 | 0.030642 | 0.16768 | MYH10\|RAB35\|RASA1 | http://amigo.geneontology.org/amigo/term/GO:0000281 |
| protein stabi-lization | Gene Ontol-ogy | GO:0050821 | 6 | 183 | 0.030647 | 0.16768 | ZNF207\|NAA15\|OTUD3\|PTEN\|MDM4\|USP36 | http://amigo.geneontology.org/amigo/term/GO:0050821 |
| regulation of lipid meta-bolic process | Gene Ontol-ogy | GO:0019216 | 4 | 92 | 0.031449 | 0.171339 | PEX11A\|PPARD\|TGS1\|TBL1XR1 | http://amigo.geneontology.org/amigo/term/GO:0019216 |
| positive regulation of dendrite morphogen-esis | Gene Ontol-ogy | GO:0050775 | 2 | 21 | 0.032486 | 0.171585 | PARP6\|CAPRIN1 | http://amigo.geneontology.org/amigo/term/GO:0050775 |
| phosphati-dylserine acyl-chain remodeling | Gene Ontol-ogy | GO:0036150 | 2 | 21 | 0.032486 | 0.171585 | MBOAT2\|PLA2G4D | http://amigo.geneontology.org/amigo/term/GO:0036150 |
| neural crest cell devel-opment | Gene Ontol-ogy | GO:0014032 | 2 | 21 | 0.032486 | 0.171585 | EDNRA\|TAPT1 | http://amigo.geneontology.org/amigo/term/GO:0014032 |
| thyroid gland development | Gene Ontol-ogy | GO:0030878 | 2 | 21 | 0.032486 | 0.171585 | SHH\|FGF8 | http://amigo.geneontology.org/amigo/term/GO:0030878 |
| dopaminer-gic neuron differentia-tion | Gene Ontol-ogy | GO:0071542 | 2 | 21 | 0.032486 | 0.171585 | SHH\|FGF8 | http://amigo.geneontology.org/amigo/term/GO:0071542 |
| brain mor-phogenesis | Gene Ontol-ogy | GO:0048854 | 2 | 21 | 0.032486 | 0.171585 | PTEN\|FBXW11 | http://amigo.geneontology.org/amigo/term/GO:0048854 |
| negative regulation of cardiac mus-cle cell apoptotic process | Gene Ontol-ogy | GO:0010667 | 2 | 21 | 0.032486 | 0.171585 | PPP1R10\|HEY2 | http://amigo.geneontology.org/amigo/term/GO:0010667 |
| regulation of smoothened signaling pathway | Gene Ontol-ogy | GO:0008589 | 2 | 21 | 0.032486 | 0.171585 | C2CD3\|PHIP | http://amigo.geneontology.org/amigo/term/GO:0008589 |
| exocytosis | Gene Ontol-ogy | GO:0006887 | 4 | 93 | 0.032493 | 0.171585 | MYH10\|CADPS\|SYTL1\|EXOC6B | http://amigo.geneontology.org/amigo/term/GO:0006887 |
| cytoskeleton organization | Gene Ontol- | GO:0007010 | 5 | 138 | 0.032834 | 0.172845 | BRWD3\|BRWD1\|ZRANB1\|ZMYM4\|PHIP | http://amigo.geneontolo- |

| | | | | | | | | |
|---|---|---|---|---|---|---|---|---|
| | ogy | | | | | | | gy.org/amigo/term/GO:0007010 |
| cell division | Gene Ontology | GO:0051301 | 9 | 346 | 0.033616 | 0.176074 | ZNF207\|CSNK1A1\|NEK1\|STAG2\|KATNB1\|PDS5A\|POGZ\|RNF8\|ANAPC1 | http://amigo.geneontology.org/amigo/term/GO:0051301 |
| lipid metabolic process | Gene Ontology | GO:0006629 | 5 | 140 | 0.034547 | 0.180219 | PTPRN2\|PPARD\|MBD1\|NR1H2\|PAFAH1B2 | http://amigo.geneontology.org/amigo/term/GO:0006629 |
| Wnt signaling pathway | Gene Ontology | GO:0016055 | 6 | 189 | 0.034894 | 0.180258 | FBXW11\|ZRANB1\|RYK\|DKK4\|USP34\|CSNK1A1 | http://amigo.geneontology.org/amigo/term/GO:0016055 |
| embryonic digit morphogenesis | Gene Ontology | GO:0042733 | 3 | 55 | 0.035085 | 0.180258 | SHH\|C2CD3\|HOXD13 | http://amigo.geneontology.org/amigo/term/GO:0042733 |
| double-strand break repair via nonhomologous end joining | Gene Ontology | GO:0006303 | 3 | 55 | 0.035085 | 0.180258 | KDM2A\|BRCA1\|RNF8 | http://amigo.geneontology.org/amigo/term/GO:0006303 |
| nuclear-transcribed mRNA poly(A) tail shortening | Gene Ontology | GO:0000289 | 2 | 22 | 0.035155 | 0.180258 | CNOT8\|CNOT4 | http://amigo.geneontology.org/amigo/term/GO:0000289 |
| retina development in camera-type eye | Gene Ontology | GO:0060041 | 3 | 56 | 0.036636 | 0.186953 | MYH10\|MAX\|PDE6A | http://amigo.geneontology.org/amigo/term/GO:0060041 |
| positive regulation of cold-induced thermogenesis | Gene Ontology | GO:0120162 | 4 | 97 | 0.036869 | 0.18777 | OGT\|ADRB3\|TRPM8\|KDM3A | http://amigo.geneontology.org/amigo/term/GO:0120162 |
| transcription by RNA polymerase III | Gene Ontology | GO:0006383 | 2 | 23 | 0.037906 | 0.190415 | SNAPC2\|ZNF345 | http://amigo.geneontology.org/amigo/term/GO:0006383 |
| positive regulation of apoptotic signaling pathway | Gene Ontology | GO:2001235 | 2 | 23 | 0.037906 | 0.190415 | MAPK9\|TRAF7 | http://amigo.geneontology.org/amigo/term/GO:2001235 |
| bone resorption | Gene Ontology | GO:0045453 | 2 | 23 | 0.037906 | 0.190415 | ZNF675\|NCDN | http://amigo.geneontology.org/amigo/term/GO:0045453 |
| positive regulation of mRNA splicing, via spliceosome | Gene Ontology | GO:0048026 | 2 | 23 | 0.037906 | 0.190415 | TRA2B\|RBMX | http://amigo.geneontology.org/amigo/term/GO:0048026 |
| positive regulation of exocytosis | Gene Ontology | GO:0045921 | 2 | 23 | 0.037906 | 0.190415 | RAB9A\|CADPS | http://amigo.geneontology.org/amigo/term/GO:0045921 |
| steroid hormone mediated signaling pathway | Gene Ontology | GO:0043401 | 2 | 23 | 0.037906 | 0.190415 | PPARD\|NR2C1 | http://amigo.geneontology.org/amigo/term/GO:0043401 |

| | | | | | | | | |
|---|---|---|---|---|---|---|---|---|
| positive regulation of canonical Wnt signaling pathway | Gene Ontology | GO:0090263 | 5 | 145 | 0.039068 | 0.195872 | DLX5\|JRK\|USP34\|PSMD10\|TBL1XR1 | http://amigo.geneontology.org/amigo/term/GO:0090263 |
| peptidyl-tyrosine dephosphorylation | Gene Ontology | GO:0035335 | 4 | 99 | 0.039178 | 0.196044 | PTPRN2\|PTEN\|DUSP11\|DUSP26 | http://amigo.geneontology.org/amigo/term/GO:0035335 |
| posttranscriptional regulation of gene expression | Gene Ontology | GO:0010608 | 2 | 24 | 0.040736 | 0.201876 | PUM2\|ZC3H7B | http://amigo.geneontology.org/amigo/term/GO:0010608 |
| low-density lipoprotein particle clearance | Gene Ontology | GO:0034383 | 2 | 24 | 0.040736 | 0.201876 | POGZ\|AP2A1 | http://amigo.geneontology.org/amigo/term/GO:0034383 |
| nervous system development | Gene Ontology | GO:0007399 | 8 | 305 | 0.041944 | 0.207468 | CHD2\|RYK\|LY6H\|NR2C2\|PURA\|GUSBP1\|DYRK1A\|DLX5 | http://amigo.geneontology.org/amigo/term/GO:0007399 |
| phospholipase C-activating G protein-coupled receptor signaling pathway | Gene Ontology | GO:0007200 | 3 | 60 | 0.043185 | 0.210611 | LTB4R\|FPR2\|ADRA1B | http://amigo.geneontology.org/amigo/term/GO:0007200 |
| methylation | Gene Ontology | GO:0032259 | 3 | 60 | 0.043185 | 0.210611 | N6AMT1\|COMTD1\|NSUN7 | http://amigo.geneontology.org/amigo/term/GO:0032259 |
| positive regulation of GTPase activity | Gene Ontology | GO:0043547 | 8 | 307 | 0.04326 | 0.210611 | RAB3GAP1\|VAV3\|ARHGAP18\|NF1\|RGPD5\|FAM13A\|RASA1\|BNIP2 | http://amigo.geneontology.org/amigo/term/GO:0043547 |
| positive regulation of innate immune response | Gene Ontology | GO:0045089 | 2 | 25 | 0.043642 | 0.210611 | FPR2\|POLR3C | http://amigo.geneontology.org/amigo/term/GO:0045089 |
| positive regulation of cell cycle arrest | Gene Ontology | GO:0071158 | 2 | 25 | 0.043642 | 0.210611 | BRCA1\|TP73 | http://amigo.geneontology.org/amigo/term/GO:0071158 |
| retina layer formation | Gene Ontology | GO:0010842 | 2 | 25 | 0.043642 | 0.210611 | SDK2\|CHD2 | http://amigo.geneontology.org/amigo/term/GO:0010842 |
| phosphatidylethanolamine acyl-chain remodeling | Gene Ontology | GO:0036152 | 2 | 25 | 0.043642 | 0.210611 | MBOAT2\|PLA2G4D | http://amigo.geneontology.org/amigo/term/GO:0036152 |
| negative regulation of osteoclast differentiation | Gene Ontology | GO:0045671 | 2 | 25 | 0.043642 | 0.210611 | ZNF675\|NF1 | http://amigo.geneontology.org/amigo/term/GO:0045671 |
| phospholipid translocation | Gene Ontology | GO:0045332 | 2 | 25 | 0.043642 | 0.210611 | TMEM30B\|ATP9B | http://amigo.geneontology.org/amigo/term/GO:0045332 |

| | | | | | | | | |
|---|---|---|---|---|---|---|---|---|
| regulation of rhodopsin mediated signaling pathway | Gene Ontol-ogy | GO:0022400 | 2 | 26 | 0.046624 | 0.219438 | CNGB1\|PDE6A | http://amigo.geneontolo-gy.org/amigo/term/GO:0022400 |
| positive regulation of protein inser-tion into mitochondri-al membrane involved in apoptotic signaling pathway | Gene Ontol-ogy | GO:1900740 | 2 | 26 | 0.046624 | 0.219438 | TP73\|TFDP1 | http://amigo.geneontolo-gy.org/amigo/term/GO:1900740 |
| positive regulation of muscle cell differentia-tion | Gene Ontol-ogy | GO:0051149 | 2 | 26 | 0.046624 | 0.219438 | MEF2A\|BNIP2 | http://amigo.geneontolo-gy.org/amigo/term/GO:0051149 |
| negative regulation of stress fiber assembly | Gene Ontol-ogy | GO:0051497 | 2 | 26 | 0.046624 | 0.219438 | ARHGEF18\|PHLDB2 | http://amigo.geneontolo-gy.org/amigo/term/GO:0051497 |
| pigmentation | Gene Ontol-ogy | GO:0043473 | 2 | 26 | 0.046624 | 0.219438 | NF1\|MYSM1 | http://amigo.geneontolo-gy.org/amigo/term/GO:0043473 |
| regulation of actin fila-ment polymeriza-tion | Gene Ontol-ogy | GO:0030833 | 2 | 26 | 0.046624 | 0.219438 | RASA1\|ARHGAP18 | http://amigo.geneontolo-gy.org/amigo/term/GO:0030833 |
| vasculogene-sis | Gene Ontol-ogy | GO:0001570 | 3 | 62 | 0.046664 | 0.219438 | SHH\|HEY2\|RASA1 | http://amigo.geneontolo-gy.org/amigo/term/GO:0001570 |
| somatic stem cell popula-tion mainte-nance | Gene Ontol-ogy | GO:0035019 | 3 | 63 | 0.048453 | 0.227026 | VANGL2\|CDX2\|FOXP1 | http://amigo.geneontolo-gy.org/amigo/term/GO:0035019 |
| visual per-ception | Gene Ontol-ogy | GO:0007601 | 6 | 206 | 0.048916 | 0.228778 | BBS9\|NYX\|CNGB1\|SLC24A1\|CLN6\|PDE6A | http://amigo.geneontolo-gy.org/amigo/term/GO:0007601 |
| bone mor-phogenesis | Gene Ontol-ogy | GO:0060349 | 2 | 27 | 0.049678 | 0.229425 | LRP5L\|SFTPC | http://amigo.geneontolo-gy.org/amigo/term/GO:0060349 |
| coronary vasculature development | Gene Ontol-ogy | GO:0060976 | 2 | 27 | 0.049678 | 0.229425 | MYH10\|DCTN5 | http://amigo.geneontolo-gy.org/amigo/term/GO:0060976 |

**Table S2. Binding residues predicted by molecular docking of the target to the drug.**

| **Term** | **Residue** | **Length（Å）** |
|---|---|---|
| DDX17_Demecolcine | Demecolcine_GLN-180<br>Demecolcine_ASP-404 | 2.6<br>2.0 |
| DDX17_TestosteroneEnanthate | TestosteroneEnanthate_PHE-94<br>TestosteroneEnanthate_LYS-76 | 2.1<br>2.1 |
| SF3B1_TestosteroneEnanthate | SF3B1_TestosteroneEnanthate_ASP-511 | 2.3 |

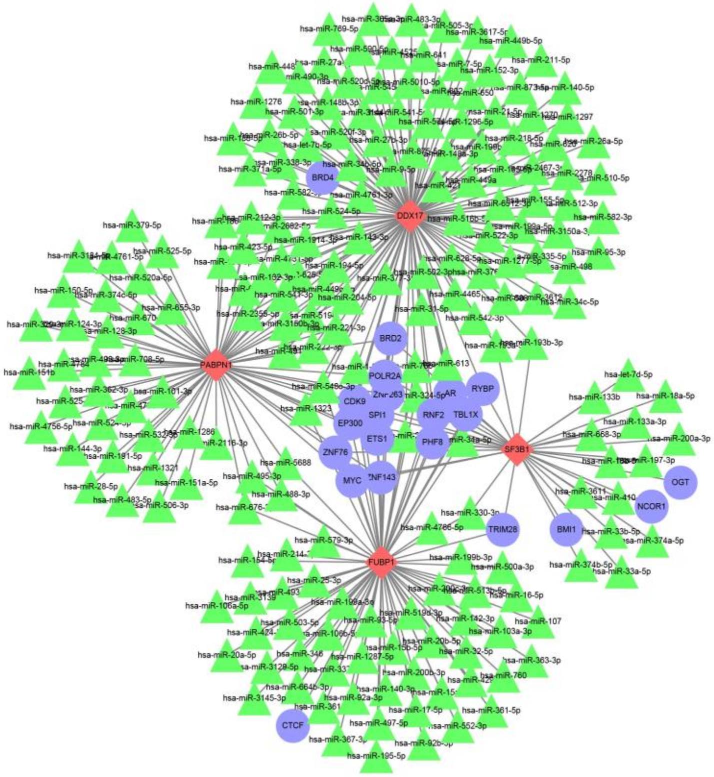


**Fig. S1 Transcription factors (TFs) regulatory network of 4 biomarkers**
Notes: The red diamond represents 4 biomarkers, the blue circle represents TFs, and the green triangle represents miRNA.
